# The 3D theory of osseointegration: material, topography, and time as interdependent determinants of bone–implant integration

**DOI:** 10.1186/s40729-025-00639-1

**Published:** 2025-08-02

**Authors:** Takahiro Ogawa, Makoto Hirota, Rune Shibata, Takanori Matsuura, Keiji Komatsu, Juri Saruta, Wael Att

**Affiliations:** 1 Weintraub Center for Reconstructive Biotechnology, UCLA School of Dentistry, Los Angeles, CA USA; 2 Division of Regenerative and Reconstructive Sciences, UCLA School of Dentistry, Los Angeles, CA USA; 3 Division of Oral and Maxillofacial Surgery, Kyoto University Graduate School of Medicine, Kyoto, Japan; 4 Department of Periodontology, Graduate School of Medical and Dental Sciences, Institute of Science Tokyo, Tokyo, Japan; 5 Department of Lifetime Oral Health Care Sciences, Graduate School of Medical and Dental Sciences, Institute of Science Tokyo, Tokyo, Japan; 6 Division of Education Planning, School of Dentistry, Kanagawa Dental University, Yokosuka, Japan; 7 Medical Center - University of Freiburg, Center for Dental Medicine, Department of Prosthetic Dentistry, Faculty of Medicine, University of Freiburg, Freiburg, Germany; 8 Weintraub Center for Reconstructive Biotechnology, Division of Regenerative and Reconstructive Sciences, UCLA School of Dentistry, 10833 Le Conte Avenue, B3-087, Box951668, Los Angeles, CA 90095-1668 USA

## Abstract

Despite widespread clinical success of dental implants, several fundamental questions remain unresolved: How does osseointegration—a biological phenomenon distinct from conventional bone healing—actually occur? Why does bone–implant contact never reach 100%? Why has there been minimal innovation in commercial implant surfaces over the past three decades? And why has the failure rate plateaued at around 8%? This review introduces the 3D Theory of Osseointegration, which conceptualizes implant integration as governed by three interdependent and dynamic determinants: material composition (Dimension 1), surface topography/roughness (Dimension 2), and time, which critically influences the physicochemical properties of implant surfaces (Dimension 3). For Dimension 1, the biocompatibility of various metals has been extensively studied, with commercially pure titanium and titanium alloys firmly established as the gold standard for dental implants. Dimension 3 underscores the long-overlooked impact of time—specifically, the biological aging of titanium surfaces caused by hydrocarbon accumulation and the loss of hydrophilicity—which significantly diminishes osteoconductivity. Importantly, recent studies have uncovered that this time-dependent degradation, once seen as an inevitable limitation, is in fact fully reversible. UV photofunctionalization restores surface hydrophilicity and removes hydrocarbon contaminants, revitalizing the bioactivity of titanium. This breakthrough not only resolves a long-standing barrier to optimal osseointegration but also establishes quantitative thresholds for key physicochemical parameters—such as carbon content and surface wettability. As a result, Dimensions 1 and 3—material and physicochemical properties—are approaching maturity in terms of optimization. In contrast, Dimension 2, surface topography, remains relatively underdeveloped despite decades of research and the clinical success of microrough surfaces. Now that UV photofunctionalization effectively mitigates biological aging and unlocks the full physicochemical potential of implant surfaces, the advancement of surface topography becomes the next critical frontier. This review critically examines each dimension, their interactions, and the limitations of current topographical design. It advocates for a shift from empirical to mechanism-driven engineering of implant surfaces and underscores the need for intentional synergy across all three dimensions. The 3D Theory of Osseointegration offers a structured framework to inform future implant design and research, aiming to better control and optimize the biological process of integration while acknowledging the complexities that still remain to be fully addressed.

## Introduction

Metallic implants have revolutionized the fields of orthopedic, dental, and craniofacial rehabilitation, offering reliable solutions to restore function and aesthetics through endosseous or transmucosal anchorage. The biological success of these implants depends on a phenomenon known as osseointegration, which refers to the direct and stable integration of the implant surface with surrounding bone tissue [1]. Traditionally, osseointegration has been viewed as a function of two principal factors: the type of implant material and its surface topography or roughness. Advances in biomaterials and surface engineering have made significant strides in optimizing these parameters, with materials such as commercially pure titanium (cp Titanium) and titanium alloys receiving wide clinical acceptance. Likewise, micro-scale surface modifications—ranging from sandblasting to acid-etching—have greatly enhanced the biological and mechanical integration of implants [2–7].

However, a critical third factor has recently gained recognition: time. Titanium surfaces undergo biological aging— time-dependent physicochemical degradation marked by the loss of hydrophilicity and the accumulation of hydrocarbon contaminants, even under sterile storage conditions [8–10]. These unintended changes significantly compromise the osteoconductive capacity of titanium. In contrast to material composition and surface topography, which are static and fixed characteristics, the time factor introduces a dynamic and evolving variable. This newly identified dimension has profound implications for understanding and optimizing implant performance. Most importantly, it shifts attention beyond *what* an implant is made of and *how* its surface is crafted—to also include *when* the implant is used [11–13].

**Fig. 1:**
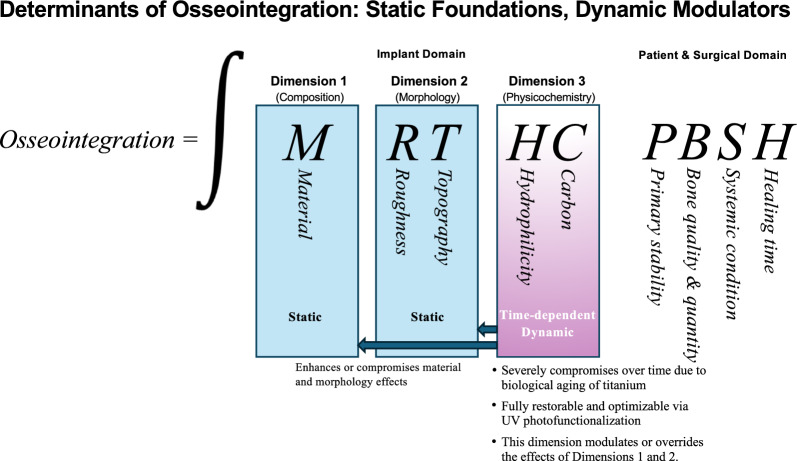
The three dimensions determining osseointegration. This figure illustrates the three core dimensions within the implant domain that govern osseointegration: material composition (Dimension 1), surface topography or roughness (Dimension 2), and time-sensitive physicochemical properties (Dimension 3). While Dimensions 1 and 2 are traditionally considered static and fixed, recent discoveries have revealed that Dimension 3 is dynamic and evolves over time. This time-dependent transformation—referred to as biological aging of implant surfaces—is marked by hydrocarbon accumulation and a loss of surface hydrophilicity. This paradigm shift highlights that Dimension 3 not only changes independently but can also alter the functional expression of Dimensions 1 and 2, thereby redefining the science and biology of implants

Although overall implant success is inherently multifactorial, requiring the coordinated integration of surgical planning, prosthetic execution, clinician expertise, and host conditions, successful osseointegration depends on implant-related and patient/surgical factors, as outlined in Fig. 1. Among these, the implant domain exerts a decisive biological influence. This review introduces the 3D Theory of Osseointegration, in which material (Dimension 1), surface topography/roughness (Dimension 2), and time-dependent physicochemical properties (Dimension 3) interact to shape the biological effectiveness of implants. Although these three dimensions are often considered independently, they are in fact interrelated—sometimes functioning independently, sometimes interdependently, and at times synergistically. Understanding and optimizing each dimension, both individually and collectively, is critical for the advancement of implant therapy. While technologies such as UV photofunctionalization have demonstrated efficacy in reversing biological aging [14–17], the primary aim of this review is to contextualize the time factor within the broader framework of implant science. We critically examine the evidence supporting each dimension and propose a unified model for evaluating and designing implant surfaces for future development, testing, and clinical application. Given the scientific focus on uncovering the mechanisms of osseointegration and addressing unresolved questions, this review emphasizes in vitro cellular and preclinical animal studies.

## The pursuit of complete osseointegration

Despite decades of advancements, a critical biological reality remains unchanged in implant dentistry. Persistent clinical challenges continue to hinder outcomes, including: prolonged healing periods required to achieve osseointegration [12, 18]; restricted indications for implant therapy due to local and systemic host conditions [19–23]; and a failure rate that has remained stagnant at approximately 8% [24–26]. These challenges are partly attributable to the suboptimal bone-implant contact (BIC) achieved with currently available implants. Even modern microrough titanium implants, widely used in clinical practice, demonstrate a BIC ranging from 45 to 65% [27–31]—a figure far below the theoretical ideal of 100%. This gap underscores a fundamental limitation: osseointegration, as it is currently pursued, has reached a plateau.

Why BIC fails to reach 100% has long been a mystery in implant science [13]. Lacking a definitive answer, implant surface technology has seen limited innovation over the past three decades [32, 33]. The question—why full osseointegration is not achieved—has either been overlooked or remained unaddressed. Historically, the definition of osseointegration emphasized areas of bone formation in direct contact with titanium, while largely ignoring the presence of soft tissue encapsulation or voids where bone fails to integrate. Even in animal models, which are often presumed to show superior bone healing than humans, up to 25% of the microrough implant surface can be covered or intervened by soft tissue instead of bone [34].

**Fig. 2:**
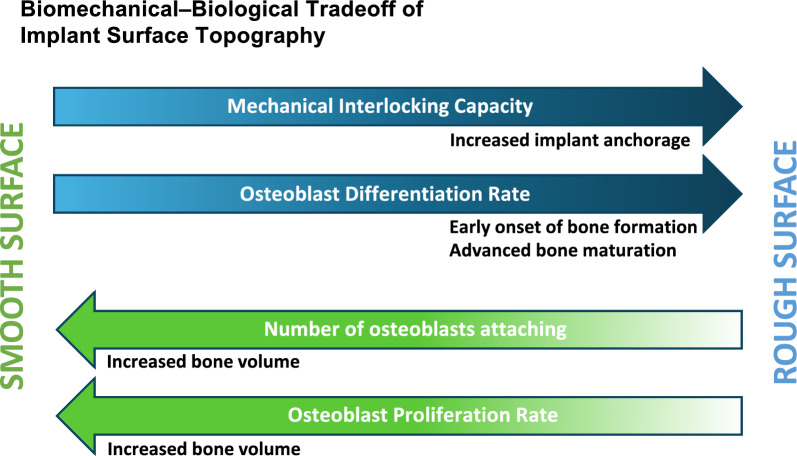
Biomechanical–biological tradeoff of implant surface topography. Implant surface roughness presents a functional tradeoff between mechanical and biological outcomes. Rougher surfaces enhance mechanical interlocking with bone, providing superior biomechanical anchorage. However, at the cellular level, osteoblasts demonstrate an inverse relationship between proliferation and differentiation—when differentiation is enhanced, proliferation is suppressed, and vice versa. Rough surfaces promote osteoblast differentiation, accelerating the initiation of osseointegration, but concurrently reduce proliferation rates and initial cell attachment. As a result, although osseointegration may be established more quickly, the total volume of newly formed bone tends to be lower on rough surfaces compared to smoother, machined ones. This inherent biomechanical–biological tradeoff has posed a persistent challenge in implant surface design and osseointegration science, underscoring the need for innovations that can reconcile both strength and biology

This stagnation may be closely related to the biological response of osteoblasts to microrough surfaces. Current microrough titanium surfaces—used in most commercial dental implants—have not been fundamentally redesigned in decades [35, 36]. Although increased surface roughness promotes osteoblastic differentiation, it simultaneously suppresses cellular proliferation, as illustrated in Fig. 2 [34, 37–42]. The biological limitations imposed by these surfaces may explain the consistent shortfall in achieving complete osseointegration.

## Dimension 1—material: foundation of biocompatibility

### Commercially pure titanium (cpTi) vs. titanium alloy (Ti-6Al-4V)

#### Background

Titanium remains the material of choice for dental and orthopedic implants due to its high corrosion resistance, favorable mechanical properties, and excellent long-term biocompatibility. Since Brånemark’s seminal work on osseointegration, titanium has been unrivaled in clinical success [1, 43]. Its formation of a stable, self-passivating oxide layer (TiO₂) enables it to coexist with surrounding tissue without eliciting severe inflammatory responses. However, titanium’s biocompatibility should not be mistaken for true bioactivity. While it passively supports bone apposition, titanium is fundamentally bioinert—lacking the ability to actively signal or stimulate host cellular responses [44]. This distinction is critical in understanding the limitations of current implant materials, especially in suboptimal biological environments. Moreover, the fact that only 45–65% of the implant surface is typically in direct contact with bone—as consistently observed in histological analyses—suggests that a substantial portion of the implant may not achieve true osseointegration, raising the possibility that parts of the titanium surface function not even as bioinert, but as biotolerant—merely tolerated by the body without integration.

Titanium used in medical and dental applications exists in two major forms: commercially pure titanium (cpTi) and titanium alloys. Commercially pure titanium (cpTi) includes both industrial and medical grades. ASTM F67 is a standard specification for unalloyed titanium and titanium alloy for surgical implant applications (medical grade). Medical-grade commercially pure titanium (cpTi, ASTM F67) contains 99% or more titanium by weight and is classified into four grades (Grades 1 to 4) according to the American Society for Testing and Materials (ASTM F67). This classification is based primarily on the oxygen and iron content. Grade 1 titanium has the highest purity, with the lowest oxygen (0.18%) and iron content, resulting in the highest corrosion resistance but the lowest mechanical strength. Grade 4, in contrast, contains up to 0.4% oxygen, which increases strength but slightly reduces corrosion resistance.

To improve mechanical strength for load-bearing applications, titanium is often alloyed with other elements. The most widely used alloy in biomedical settings is Ti-6Al-4V ELI (Extra Low Interstitials), classified as Grade 23 under ASTM F136. This high-purity variant of the more common Grade 5 (Ti-6Al-4V) was specifically developed for enhanced biocompatibility in medical implants. The alloy consists of approximately 90% titanium, 6% aluminum, and 4% vanadium, with tightly controlled levels of interstitial elements such as iron (≤0.25%) and oxygen (≤0.2%). Aluminum serves to stabilize the alpha phase and reduce the density, while vanadium contributes to beta phase stabilization and enhances thermal and mechanical stability. Titanium alloys are classified from Grade 5 to Grade 29 under various ASTM standards, each optimized for different clinical and mechanical demands [32].

The regulatory classification of titanium materials is well established. In the United States, cpTi and Ti-6Al-4V used in medical implants must comply with FDA-recognized consensus standards. The biocompatibility of these materials has been extensively evaluated under ISO 10993 and is widely accepted worldwide. The choice between cpTi and Ti-6Al-4V in clinical settings is often application-specific. Dental implants predominantly use cpTi, especially Grade 4, due to its favorable balance of strength and corrosion resistance for intraoral environments [32]. In contrast, orthopedic implants, which often require greater load-bearing capability, frequently use Ti-6Al-4V ELI.

It is important to note that surface topography—particularly at the micro-scale—plays a pivotal role in determining the extent and quality of osseointegration. However, achieving identical surface morphologies across different titanium materials is inherently challenging. For example, applying the same surface modification methods—such as acid etching or sandblasting—to commercially pure titanium (cpTi) and Ti-6Al-4V ELI can yield markedly different outcomes due to their distinct alloy compositions and mechanical properties. Ti-6Al-4V ELI exhibits greater hardness and chemical resistance than cpTi, resulting in reduced surface reactivity. Specifically, its higher activation energy in acidic environments makes it less susceptible to etching, thereby producing a less defined microrough surface compared to cpTi [45]. As a result, Ti-6Al-4V ELI surfaces tend to support lower levels of bone formation, attributed to their diminished microroughness. These differences highlight the complex interplay between material chemistry and surface topography and underscore the need for careful interpretation when comparing biological responses across titanium-containing materials.

To ensure meaningful comparisons, this chapter will focus primarily on studies using cpTi and Ti-6Al-4V implants with machined (smooth) surfaces, where surface differences are minimized. Studies involving roughened surfaces will be included when quantitative surface roughness parameters (e.g., Ra, Sa, Rz) are provided, allowing assessment of whether surface topography might confound the biological outcomes. When materials with different inherent surface textures are compared, special attention is paid to those surface parameters, as they can significantly influence cell behavior, protein adsorption, and bone integration.

#### Biomechanical and histological evaluation

Numerous studies have compared commercially pure titanium (cpTi) and titanium alloy (Ti-6Al-4V) in terms of their osseointegration potential. While mechanical performance is a major determinant in clinical material selection, especially in orthopedic applications, the biological compatibility—specifically, the material’s ability to support and maintain bone–implant integration—is a critical factor that requires independent evaluation.

In a rabbit femur and tibia model, experimental implants made of cpTi and Ti-6Al-4V with machined surfaces and comparable surface roughness (Sa ≈ 0.5–0.6 µm) were evaluated for biomechanical anchorage and histological integration [46]. After 16 weeks of healing, removal torque values were approximately 25% higher for cpTi implants compared to Ti-6Al-4V implants. Interestingly, histomorphometric parameters such as bone length and bone area did not show significant differences between the two materials. This discrepancy suggests the presence of unknown biological factors influencing the biomechanical strength of osseointegration beyond what is captured by histology alone.

In another study, sandblasted screw-shaped cpTi and Ti-6Al-4V implants were placed into rabbit tibiae [47]. Both implant types had a similar surface roughness (Sa ≈ 1.1–1.2 µm). After 3 months of healing, removal torque values were again higher for cpTi implants. Histological observations did not reveal a significant difference between the groups, although a slightly higher bone volume was noted around Ti-6Al-4V implants. A similar experiment using machined cpTi and Ti-6Al-4V implants with no significant difference in surface roughness parameters demonstrated that, after 6 months of healing, removal torque was 25% higher for cpTi implants [48]. Additionally, cpTi showed a higher BIC, although bone volume was comparable between the two.

In a miniature pig model, implants with sandblasted and acid-etched surfaces were placed in the maxilla [45]. Ti-6Al-4V implants exhibited a higher Sa value. BIC increased progressively up to week 8 for cpTi implants, while BIC for Ti-6Al-4V peaked at week 2 and declined thereafter. At week 8, the BIC was 2.5 times higher for cpTi implants than Ti-6Al-4V. A recent review summarized that the biomechanical strength of osseointegration is generally higher for cpTi implants than Ti-6Al-4V implants. However, no consistent evidence supports a significant difference in bone formation between the two materials [49].

It is worth noting that the magnitude and consistency of differences observed in biomechanical assessments between cpTi and Ti-6Al-4V implants tend to exceed those reported in histological analyses. Histological interpretation is subject to numerous confounding factors, including embedding techniques, distortion of the tissue–material interface during sectioning, variability in grinding methods, sample orientation, selection and definition of morphometric parameters, and the inherent subjectivity of image interpretation. While histology remains the gold standard for evaluating bone–implant interfaces, biomechanical testing—by directly measuring implant anchorage—offers a more functionally relevant endpoint, particularly for load-bearing applications. Moreover, biomechanical studies typically employ larger sample sizes and more standardized protocols, which may provide greater statistical power and reproducibility compared to histological assessments. Importantly, as previously discussed, subtle but meaningful differences in surface topography—such as feature configuration, randomness, and structural density—between cpTi and Ti-6Al-4V ELI may influence biological responses, even when reported quantitative roughness values appear similar. These microarchitectural variations must be taken into account when interpreting results, despite efforts in the present review to compare the two materials using machined or surface-modified specimens with nominally equivalent roughness metrics.

#### Cellular response

The behavior of osteogenic cells on implant materials is critical to understanding the biological mechanisms underlying histological and biomechanical outcomes. A well-controlled in vitro study using MG63 osteoblast-like cells compared the cellular response to cp titanium and Ti-6Al-4V alloy [50]. Both materials had polished surfaces with comparable average roughness (Sa ≈ 0.2 µm). The study revealed that cell proliferation was approximately 70% higher on cp titanium. However, markers of osteogenic differentiation—including transforming growth factor-beta expression, collagen production, and osteocalcin secretion—showed no significant differences between the two materials.

Concerns have been raised regarding the potential ion release from Ti-6Al-4V, particularly vanadium and aluminum. These ions have been detected outside of implant structures in ground histological sections, suggesting in vivo leakage [51]. In vitro, ions released from Ti-6Al-4V have been shown to inhibit the differentiation of rat bone marrow-derived mesenchymal stem cells into osteoblasts [52]. Notably, these ions do not appear to impair cell proliferation, but rather interfere with osteogenic differentiation—especially at its later stages. Other studies have similarly identified vanadium ions as inhibitors of osteoblastic mineralization [52].

In addition to effects on osteoblasts, Ti-6Al-4V has been shown to provoke inflammatory responses. Exposure of macrophages to Ti-6Al-4V alloy induced the release of pro-inflammatory mediators, including prostaglandin E2, interleukin-1 (IL-1), tumor necrosis factor (TNF), and interleukin-6 (IL-6) [53, 54]. These inflammatory signals may in turn activate fibroblasts and contribute to osteolysis through chronic inflammation [55].

A systematic review focusing on in vitro studies—primarily involving fibroblasts—concluded that, when Ti-6Al-4V is subjected to corrosion or wear (conditions that promote particle release), the resulting exposure to aluminum and vanadium ions exhibits a greater cytotoxic effect compared to cp titanium [56]. Lastly, it is important to note that the cytotoxic and inflammatory effects of Ti-6Al-4V are influenced by several factors, including the size, concentration, and chemical composition of the released particles and debris [57]. The mutagenic potential associated with long-term exposure to these ions remains a subject of ongoing investigation.

#### Summary

Across multiple preclinical studies, commercially pure titanium (cpTi) consistently demonstrates superior biomechanical integration compared to Ti-6Al-4V, despite both materials often exhibiting similar bone morphometry in histological evaluations. This discrepancy suggests that factors beyond bone quantity—such as bone quality and interfacial strength between implants and bone—contribute to implant anchorage. Indeed, the degree of osteogenic differentiation can influence the stiffness and hardness of peri-implant bone and interfacial adhesion between bone and titanium [58–62]. At the cellular level, cpTi supports greater osteoblast proliferation, while Ti-6Al-4V is associated with the release of aluminum and vanadium ions that can impair osteogenic differentiation and provoke inflammatory responses. These findings collectively indicate that cpTi offers a more favorable biological environment for osseointegration, supporting its continued use in applications where biocompatibility is prioritized.

### Titanium-based materials versus CoCr alloy

Cobalt-chromium (CoCr) alloys have long been used in orthopedic implant applications due to their high mechanical strength, wear resistance, and corrosion resistance. Approved by regulatory agencies such as the FDA and classified under ASTM standards (e.g., F75), CoCr alloys are commonly found in joint prostheses and relate components but are rarely used as endosseous implants. A key distinction between titanium-based materials and CoCr alloy lies in their biological behavior: while titanium-based materials are bioinert and support direct bone bonding, CoCr alloys often rely on fibrous encapsulation and may release metal ions (e.g., cobalt and chromium) that elicit adverse cellular responses. The significantly higher modulus of elasticity of CoCr compared to bone and titanium can also contribute to stress shielding and delayed bone remodeling. Given these differences, a thorough comparison of their biomechanical, histological, and cellular responses is warranted to evaluate the suitability of CoCr for direct bone integration.

Screw-shaped cpTi and cobalt-chromium (CoCr) alloy implants were placed in rabbit tibiae [63]. Both implants had machined surfaces, though the cpTi implants exhibited more distinct traces from lathe-turning. Surface roughness parameters were not clearly reported for either material. Histomorphometric analysis revealed that the BIC was approximately 50% greater for cp titanium implants, and the removal torque values were nearly double those of the CoCr implants. Similarly, in a sheep pelvis model, CoCr implants exhibited removal torque values less than half of those seen with cp titanium implants [64]. In another study, Ti-6Al-4V and CoCr alloy implants with blasted surfaces were placed into rabbit femurs [65]. CoCr implants demonstrated significantly lower interfacial shear strength with bone, despite similar BIC values. Notably, unmineralized tissue such as cartilage and osteoid was more frequently observed around CoCr surfaces.

From a cellular perspective, the release of cobalt and chromium ions into surrounding tissues has been well documented, with corrosion by-products accumulating locally and potentially disseminating systemically [66]. Elevated levels of cobalt and chromium have been detected in the serum and urine of patients with CoCr-containing prosthetic devices [66]. In vitro studies indicate that CoCr particles exhibit higher cytotoxicity and induce more cell death than titanium-aluminum-vanadium particles, although the latter may provoke greater inflammatory cytokine production [53, 54]. Osteoblasts cultured on CoCr alloy surfaces show reduced proliferation and increased expression of proinflammatory cytokines such as interleukin-6 [67]. As mentioned earlier, the biological response to metal particles varies depending on particle size, concentration, and tissue type. It should also be noted that depending on the clinical application, cp titanium may generate more wear debris than CoCr alloys [68].

### Cp titanium vs. zirconia

#### Background

Zirconia has emerged as a potential alternative to cpTi and titanium alloys in specific areas of dental implant therapy, particularly in esthetically critical zones such as the anterior maxilla, and for patients with metal hypersensitivity or a preference for metal-free restorations. As a ceramic material, zirconia differs fundamentally from metals in surface chemistry, mechanical behavior, and biological interaction. While titanium readily forms a stable and bioactive oxide layer that supports osseointegration, zirconia’s surface is inherently less reactive. However, when properly engineered, zirconia can also support bone apposition and achieve enhanced osseointegration [41, 42, 69].

Zirconia implants are most commonly fabricated from yttria-stabilized tetragonal zirconia polycrystal (Y-TZP), which is known for its high flexural strength, fracture toughness, and resistance to low-temperature degradation. The transformation toughening mechanism of Y-TZP, where the tetragonal phase transforms to the monoclinic phase under stress, contributes to its crack resistance. To further enhance mechanical integrity and reduce internal porosity, hot isostatic pressing (HIP) is employed during the manufacturing process. HIP involves applying high pressure and temperature uniformly to the ceramic body, consolidating powders and eliminating microstructural defects, thereby improving the reliability and performance of zirconia implants [70].

Clinically, zirconia implants are often designed as one-piece systems due to challenges in creating reliable screw-retained or abutment connections that can withstand masticatory forces without compromising the material’s structural integrity. While some two-piece zirconia implant systems have been developed, they remain less common and are subject to ongoing evaluation [71].

From a regulatory standpoint, titanium implants have long-standing FDA and CE approvals with extensive clinical documentation, and they serve as the reference standard in most regulatory frameworks. Zirconia implants, although approved by regulatory agencies such as the FDA and European authorities, are relatively newer, and their long-term clinical evidence remains limited compared to titanium. Regulatory agencies often require more stringent documentation for novel materials, particularly ceramics, due to concerns over brittleness, low fracture toughness, and performance under cyclic loading.

It has been suggested—though still debated—that zirconia may be less prone to bacterial adhesion and plaque accumulation compared to titanium [72]. This chapter focuses primarily on the osseointegration potential of zirconia relative to cp titanium. As noted in prior sections, comparing titanium and zirconia implants is inherently difficult due to differences in available surface modification techniques and resulting topographies. For consistency and validity, this review emphasizes studies using machined or polished surfaces or those employing comparable surface treatments with well-characterized morphological and quantitative roughness parameters. While surface roughening is a key strategy for enhancing the osseointegration of titanium implants, modifying zirconia surfaces presents unique challenges. Traditional approaches like acid-etching or coating—effective for titanium—are not directly transferable to zirconia due to its chemical inertness and structural characteristics [73]. Moreover, increasing surface roughness through methods like sandblasting may compromise the bulk strength of zirconia by introducing potential sites for microcrack initiation and propagation. This presents an additional dilemma in zirconia surface engineering, where mechanical integrity must be balanced against biological performance. Consequently, the development of effective and safe surface modification techniques remains a critical focus in advancing zirconia-based implant therapy.

#### Histological and biomechanical evaluations

In a rat femur model, cp titanium and Y-TZP zirconia implants with machined surfaces were directly compared [70]. The average surface roughness (Ra) was 0.58 µm for cp titanium and 0.19 µm for zirconia. Despite having a smoother surface, zirconia implants demonstrated a significantly higher BIC at both 14 and 28 days of healing. Biomechanical testing via push-in force showed equivalent strength of osseointegration between the two groups. These findings suggest that the higher surface roughness of cp titanium may have compensated for its relatively lower BIC. In another rat femur study using similarly machined surfaces, zirconia implants exhibited a higher surface roughness than cp titanium implants, yet no significant differences were observed in either BIC or biomechanical strength [74].

In a pig maxilla model, implants with acid-etched surfaces were placed to compare osseointegration between titanium and zirconia [75]. Although different proprietary acid-etching protocols were used for each material, both implant types developed micro-pitted surfaces. The average surface roughness was 1.23 µm for titanium and 0.52 µm for zirconia. Despite the substantially lower roughness, zirconia implants demonstrated equivalent biomechanical strength across all three evaluated healing time points. The authors also reported reduced bone resorption at the crestal region of zirconia implants exposed to the oral cavity, potentially due to zirconia’s lower plaque affinity. A follow-up study using the same model and implants revealed a trend toward higher BIC for zirconia, though the difference was not statistically significant [76].

A rabbit tibia study provided insight into potential differences in the quality of bone integration [77]. Machined titanium and zirconia implants (with no reported roughness values) were compared. Removal torque was 80% higher for zirconia, though not statistically significant. Conversely, titanium implants had 40% greater BIC and significantly more bone area overall. Interestingly, fluorochrome labeling with calcein revealed 150% greater stained bone area for zirconia implants, suggesting denser mineralization around zirconia surfaces despite their lower BIC.

In a minipig jawbone study, surface-modified zirconia and titanium implants were evaluated after sandblasting and acid-etching [78]. Titanium implants had approximately twice the average surface roughness compared to zirconia. Removal torque testing revealed that titanium implants exhibited double the mechanical anchorage at both early and late healing stages. However, BIC was only significantly higher for titanium at the late healing time point, showing a 25% increase. A separate rabbit model study compared zirconia and titanium implants with matched micro-roughened surfaces (~1.3 µm Ra), featuring similar pore structures [79]. The two implant types demonstrated equivalent BIC and removal torque values, indicating comparable osseointegration under controlled surface conditions.

Overall, a review of the literature concluded that most animal studies report no statistically significant differences between titanium and zirconia implants regarding BIC and biomechanical retention, regardless of surface modifications [80]. These findings underscore the complex interplay of surface characteristics, material chemistry, and biological response. When appropriately engineered, zirconia demonstrates comparable osteoconductivity, positioning it as a viable alternative to titanium in implant therapy—from the perspective of osseointegration.

#### Cellular response

Human osteoblastic cell lines were cultured on cpTi and Y-TZP disks with machined surfaces, where the cp titanium exhibited higher average surface roughness [70]. These studies reported no significant differences in cell proliferation or differentiation rates between the two materials. In another study, micro-scale porous surfaces were generated on both titanium and zirconia substrates via anodic plasma oxidation [81]. Although the surfaces were qualitatively similar, quantitative roughness data were not provided. Human primary osteoblastic cells cultured on these surfaces showed comparable proliferation rates on both materials. However, the zirconia surface promoted higher expression of bone-related proteins, including bone sialoprotein and osteocalcin, suggesting enhanced osteoblastic differentiation.

Further, cp titanium and Y-TZP disks with roughened surfaces were prepared using identical mechanical protocols involving SiC papers down to 4000 mesh, resulting in equivalent surface roughness [82]. Under these conditions, osteoblastic proliferation was found to be greater on Y-TZP. Fluorescence imaging also revealed increased mineral deposition in cultures grown on zirconia, indicating enhanced matrix mineralization. A comparative study using murine pre-osteoblastic MC3T3-E1 cells reported higher initial cell viability on zirconia than on grade 2 cp titanium, despite similar surface roughness [83]. In addition, osteogenic gene expression was upregulated on zirconia surfaces, further supporting the material’s favorable cellular response [84].

#### Summary

Collectively, the current body of evidence suggests that zirconia, particularly in the form of Y-TZP, exhibits osseointegration potential comparable to that of cp titanium. Histological analyses consistently report similar or even higher BIC values for zirconia, despite its generally lower surface roughness. This observation implies that zirconia may facilitate robust bone apposition through mechanisms such as the dichotomous kinetics of osteoblast activity (see Fig. 2), or possibly through differences in surface energy or ceramic-specific interactions with the surrounding biological environment. Biomechanical assessments, including push-in and removal torque tests in various animal models, largely demonstrate comparable mechanical stability between zirconia and titanium implants. When minor differences are reported, they are typically not statistically significant and appear to reflect variations in surface characteristics rather than intrinsic material properties. In vitro cellular studies further reinforce zirconia’s biocompatibility and osteoconductive potential. Osteoblastic proliferation, viability, and expression of differentiation markers on zirconia surfaces are frequently equivalent to or greater than those observed on titanium, suggesting zirconia supports not only cell attachment and survival but also osteogenic maturation.

Despite its chemical inertness and limited amenability to conventional surface modification techniques, zirconia continues to demonstrate promising biological performance in both preclinical and in vitro settings. However, surface engineering of zirconia remains a key challenge. Efforts to increase surface roughness—an approach known to enhance osseointegration in titanium—are more difficult with zirconia due to its brittle nature and the risk of microcrack formation. Consequently, when surface-modified implants are compared, zirconia often exhibits reduced or delayed osseointegration relative to highly roughened titanium surfaces. Thus, while zirconia represents a viable and promising alternative to titanium, particularly in cases involving metal sensitivity or esthetic demand, its potential to outperform titanium in promoting long-term osseointegration remains unproven. Further well-controlled, longitudinal studies are required to determine whether zirconia can achieve or surpass the clinical performance of titanium-based implants.

### Beyond material comparison: biological impact of titanium presence in bone healing

While the comparative studies between different implant materials, such as cpTi, titanium alloys, and zirconia, provide valuable insights, it is important to recognize a more fundamental and often overlooked question in implant research: how does the presence of an implant itself alter the course of bone healing compared to natural osteotomy healing without an implant? Before evaluating the differences among various material compositions, it is essential to first understand how the biological environment responds to the introduction of a titanium implant. Several pioneering studies have attempted to address this foundational question by directly comparing osteoblastic behavior and bone wound healing with and without titanium.

High-throughput gene expression analyses, including differential display PCR [85] and DNA microarray [86], have identified gene transcripts that are uniquely upregulated during bone healing in the presence of titanium implants. These transcripts—such as apolipoprotein E, prolyl 4-hydroxylase, and others related to extracellular matrix production and bone remodeling—were not expressed in conventional osteotomy healing. The gene activation was particularly prominent during the early healing phase and was enhanced further by surface roughness. These findings indicate that the osseointegration process involves a distinct and implant-specific molecular program, separate from normal bone regeneration pathways.

Supporting this molecular evidence, in vitro studies have shown that titanium surfaces actively influence the biomechanical and structural properties of the mineralized tissue formed by osteoblasts [62]. Compared to standard polystyrene substrates, titanium promotes the formation of harder, stiffer, and more adhesive mineralized matrices. These tissues are characterized by dense, well-crystallized calcium phosphate deposition and intensive collagen organization at the tissue–implant interface, along with elevated expression of key osteogenic genes, such as collagen I and III, osteopontin, and osteocalcin. Notably, both titanium and polystyrene substrates used in these experiments exhibited super-smooth surfaces with comparable roughness, indicating that the observed differences were attributable to material composition rather than topography. Collectively, these findings emphasize that titanium presence alone orchestrates cellular function and tissue architecture in a way that fundamentally differs from bone healing without an implant.

## Dimension 2—topography/roughness: engineering biological response

### Background

Over the past four decades, a wide array of surface roughening techniques has been developed at both experimental and commercial levels. These methods—comprehensively reviewed in several publications—include acid-etching, sandblasting, titanium plasma spraying, alkaline treatment, thermal and chemical oxidation, diverse coating strategies, and combinations thereof [2, 3, 35, 36, 87]. As early as the 1980s, implant surface topography was recognized as a critical factor influencing osseointegration, though it was then often referred to more simply as “implant finish” [1]. Since that time, advances in surface science have paralleled the emergence of increasingly sophisticated topographies, accompanied by deeper biological understanding of how such surfaces mediate cell behavior and tissue integration.

Topography, by definition, refers to the form and features of a surface and can denote either the physical structures themselves or their descriptive metrics. In implant science, surface topography encompasses surface roughness, micro-irregularity, and broader morphological characteristics at multiple scales. It includes both quantitative parameters and qualitative descriptors such as sharpness, randomness, or spatial patterning [2, 3]. While the term often implies micro-scale features, implant surface topography can span from the nanoscale to the mesoscale—a range that remains below the level of gross implant geometry. Differentiating surface characteristics by scale is essential, yet such distinctions are frequently underemphasized [35]. For example, implant surfaces have been described using systematic terms such as “micro-rough and nano-smooth” to reflect multiscale features [88]. In contrast, features at the macroscopic level—such as implant threads, tapers, or platform shapes—are more appropriately categorized under the broader concept of implant design.

Understanding these dimensional layers of surface morphology is critical for evaluating how specific topographic modifications influence biological performance. As research increasingly targets engineered biological responses, the integration of both quantitative roughness metrics and qualitative spatial features is vital to fully elucidate the role of surface topography in enhancing osseointegration.

### Definitions and scales

Surface topography—often discussed interchangeably with roughness—plays a fundamental role in the biological performance of dental implants. These terms, however, should be distinguished for clarity. Roughness typically refers to the quantitative measure of surface irregularities, often expressed as average roughness or other profilometric parameters. In contrast, topography encompasses not only these quantitative values but also the qualitative characteristics of the surface, including the shape, distribution, orientation, sharpness, and randomness of surface features. Understanding both dimensions is essential to appreciating how surface design can guide cellular and tissue responses.

In the context of implant dentistry, surface topography has emerged as one of the most critical design parameters influencing osseointegration. Modifications at different dimensional scales—nano- [35, 89–93], micro- [7, 31, 36, 94–98], supra-micron [6], and meso-levels [40–42, 69]—have been developed to optimize the biological and mechanical interface between the implant and surrounding bone. Proven and hypothetical roles of features at each scale are presented in Table 1.

**Table 1:**
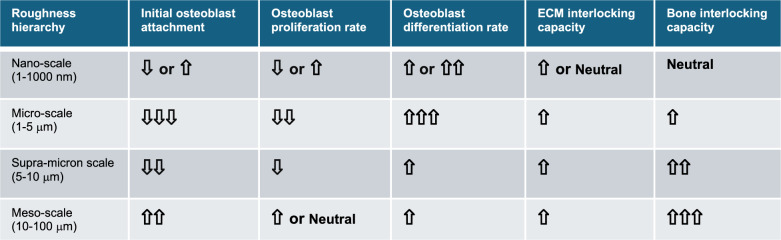
Hierarchical surface roughness and its distinct effects on cellular behavior and mechanical integration

#### Nanofeastures (1–1000 nm)

Nanofeatures influence protein adsorption and early cell signaling. While they do not significantly contribute to mechanical interlocking with bone, they play a vital role in modulating cell membrane interactions, cytoskeletal organization, gene expression, and differentiation.

#### Micro-scale features (1–5 µm)

Microtopography, often created by acid-etching, dominates the current generation of dental implant designs. These features enhance osteoblastic differentiation and promote bone anchorage by increasing surface area and enabling mechanical interdigitation with the mineralized matrix. However, the rate of osteoblastic cell proliferation is significantly reduced on these surfaces.

#### Supra-micron features (5–10 µm)

Supra-micron topography, typically produced by sandblasting, serves as an enhancement of micro-scale modifications and is implemented extensively to the current generation of implant designs. These features contribute to mechanical interlocking with bone through increased roughness and surface area.

#### Meso-scale structures (10–100 µm and beyond)

Meso-topography remains relatively underexplored due to technical challenges in fabrication. However, it is gaining increasing research interest for its potential to dramatically expand surface area, potentially enhancing the recruitment of osteogenic cells and significantly improving interlocking with bone.

### Microrough titanium surfaces: surface features, metric pitfalls, and classification gaps

**Fig. 3:**
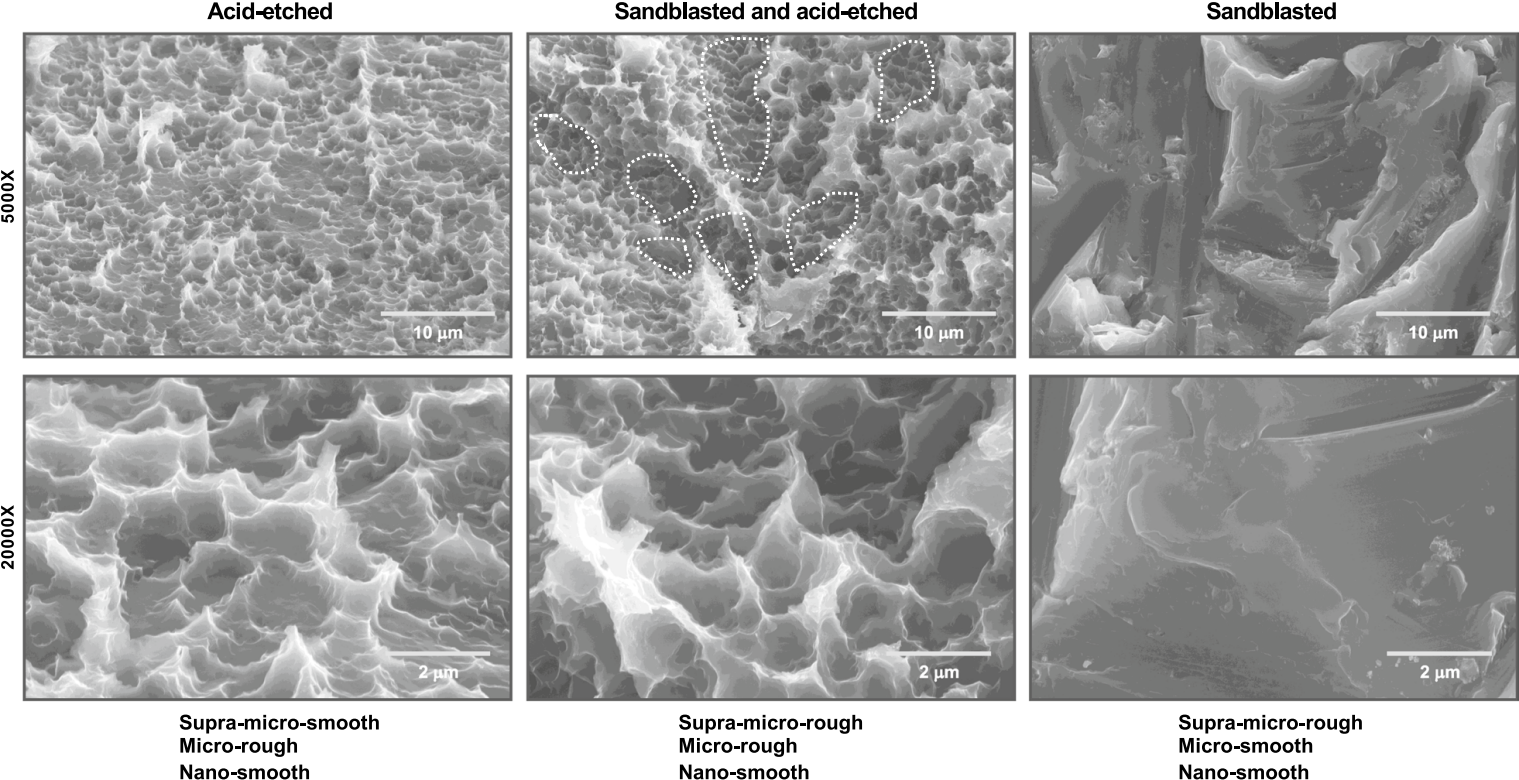
Not all roughness is equal: microroughness as the defining feature in implant surface evolution. This figure compares three titanium implant surfaces created by different modification techniques: (1) acid-etched, (2) sandblasted and acid-etched, and (3) sandblasted only. While all exhibit visible roughness, only the first two demonstrate true microroughness—a biologically active topography characterized by isotropic micro-pits and valleys that promote osteoblastic differentiation effectively. The acid-etched surface shows a homogeneous and random micro-compartmental structure, while the sandblasted and acid-etched surface combines both supra-micron irregularities (*dotted areas*) and superimposed microrough features, further enhancing biological activity. In contrast, the sandblasted-only surface, although visibly coarse, lacks defined microroughness and fails to achieve the same level of cellular interaction. This figure illustrates that roughness alone is not enough—the quality and scale of surface features are crucial. Micron-level precision, even when visually subtle, drives major differences in biological performance. In implant science, surface nuance is not trivial—it is transformative

The introduction of microrough titanium surfaces in the early to mid-1990s marked a major advancement in the field of dental implantology, substantially enhancing the potential for osseointegration. Prior to this, most commercial implants featured surfaces that were machined, sandblasted, or titanium plasma-sprayed—each possessing relatively smooth finishes without clearly defined micro-scale topography. While sandblasted and titanium plasma-sprayed surfaces presented irregularities at the supra-micro level, they lacked the compartmentalized and biologically active features later achieved through microroughening (Fig. 3).

Microrough titanium surfaces are characterized by three-dimensional features such as micro-pits or micro-compartments composed of interspersed peaks and valleys. These structures are typically isotropic—uniform in all directions—and display a random yet homogeneously distributed pattern. Among the most representative and widely used methods for generating such surfaces is acid etching, often performed with sulfuric acid, which yields the distinctive non-ordered topography associated with enhanced biological activity (Fig. 3).

From a functional perspective, microrough surfaces provide dual benefits in metallic implants used as load-bearing and anchoring devices: they enhance both mechanical interlocking and biological performance. While mechanical retention is improved through increased surface area and interfacial friction, the biological interface also benefits from enhanced cellular adhesion, differentiation, and matrix production. In implant research, biomechanical studies—typically measuring push-in strength or removal torque in vivo—evaluate the overall integration strength. However, it is important to note that few studies have isolated and quantified the purely mechanical contribution of surface topography, separate from its biological effects.

Over the years, various terms have been proposed to categorize implant surface textures—such as “smooth” [86], “rough” [99], “minimally rough” [4, 100], and “moderately rough” [4, 100]—based primarily on the Sa (average height) values. While these classifications offer a general framework, they remain conceptually and practically limited. For instance, these categories are arbitrarily bounded, lacking clear consensus on upper and lower thresholds. There is no standardized definition for what constitutes “maximum roughness,” nor is there a biologically or mechanically validated rationale for the existing ranges. In many cases, these labels are used interchangeably across different materials, techniques, or scales, contributing to significant ambiguity.

Sa is the arithmetic mean height from the mean plane and is typically derived from area-based measurements. However, Sa alone does not fully capture the nature of surface morphology. It reflects the vertical distance between the peaks and valleys but not their spatial arrangement, density, shape, or complexity. Furthermore, Sa values can be affected by numerous factors, including the type of measurement device (e.g., confocal, interferometric, or stylus profilometry), resolution threshold, scanning area, surface curvature, and software algorithms. As such, direct comparison of Sa values across different studies or systems is often unreliable and should be interpreted with caution. Additionally, these labels do not account for multiscale topography (e.g., micro- vs. nano- vs. meso-scale roughness), which is now known to influence distinct biological pathways and should be differentiated accordingly[35].

Beyond Sa, the biological behavior of osteoblasts is significantly influenced by qualitative aspects of topography. Random, irregular, and non-patterned surfaces have been shown to more effectively promote osteogenic differentiation than smooth, uniform, or highly ordered topographies [101]. In this context, acid-etched microrough titanium surfaces—with their non-ordered, randomly shaped micropits (Fig. 3)—exemplify a biologically favorable roughness profile.

**Fig. 4:**
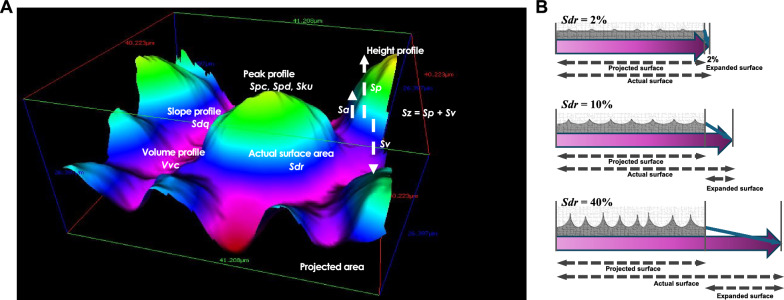
Beyond Sa: a multidimensional framework for analyzing and interpreting implant surface roughness. **A** Implant surface characterization has long relied on Sa (average height), a limited metric that captures only vertical deviations from a mean plane. However, osseointegration is governed by more than height alone. A suite of 3D surface parameters—such as Sz (maximum height), Sdr (developed interfacial area ratio), Spd (peak density), Spc (peak curvature), Sku (kurtosis: profile sharpness), Sdq (root mean square gradient: slope/inclination), and Vvc (core void volume)—collectively offer a comprehensive topographical fingerprint of implant surfaces, revealing features critical to biological responses. These metrics characterize the geometry, complexity, steepness, and spatial distribution of microfeatures, each with potential implications for osteoblast behavior, protein adsorption, and fluid interaction. **B** Sdr (developed interfacial area ratio) quantifies the relative increase in true surface area compared to a flat reference plane (projected area in panel **A**), expressed as a percentage. It reflects how much the actual surface profile has been "stretched" due to the roughness features. Among 3D surface metrics, Sdr has proven to be one of the most predictive parameters for a surface's ability to retain cells and establish mechanical interlocking. In this illustration, varying combinations of feature height and frequency yield different degrees of surface expansion. Typical machined titanium surfaces exhibit Sdr values below 2% (*top*), whereas acid-etched microrough surfaces show greater variability—with low-quality surfaces around 10% (*middle*) and high-quality surfaces reaching 40% (*bottom*). These distinctions highlight the importance of moving beyond Sa and adopting a multidimensional approach to surface design and evaluation

To more comprehensively evaluate surface topography, a variety of additional 3D roughness parameters have been introduced (Figs. 4), each offering unique insights. These include:

- Sz (maximum height): the vertical distance between the highest peak and lowest valley.
- Sdr (developed interfacial area ratio): the percentage increase in surface area relative to a flat reference plane, representing surface complexity.
- Spc (mean peak curvature): indicating sharpness of peaks.
- Spd (peak density): number of peaks per unit area.
- Sku (kurtosis): a measure of the peakedness or flatness of the surface height distribution.
- Sdq (root mean square slope): quantifying the average steepness of the surface.
- Vvc (core void volume): related to the fluid-holding capacity in the surface core.

These parameters collectively capture the geometry, spatial distribution, peak features, and intensity of surface features in three dimensions. Even among acid-etched microrough surfaces, quality variations exist based on Sdr and other 3D metrics—indicating that biologically relevant surface differences can be missed when relying on average roughness alone.

As for the above-mentioned terminology and classifications, a more robust classification system could incorporate a multivariate, metric-based approach, combining Sa with other surface parameters such as Sdr (surface area), Spd (peak density), and Sku (peak profile), thus enabling a more comprehensive and reproducible categorization. For example, surfaces could be described using standardized surface "profiles" based on a defined combination of Sa-Sdr-Spd bands, akin to how soil or rock textures are classified in geoscience. Alternatively, biologically informed categories—such as “osteoconductive-optimized,” or “bioinert”—could be considered, reflecting intended functional outcomes rather than just geometric descriptors. Thus, the traditional terminology is overly simplistic, loosely defined, and does not reflect the multidimensional nature of surface topography or its functional impact. Advancing surface classification in implant science will require standardization efforts, metric integration, and biological validation—an essential step for bridging the gap between engineering design and biological/clinical performance.

### Genuine mechanical effect

A rigorous, mechanically isolated study was conducted to investigate the effect of surface roughness on the interfacial shear strength between titanium and a PMMA-based material, used as a bone-mimicking substrate [102]. Five distinct titanium surfaces were prepared by applying combinations of sandblasting and acid-etching to machined titanium. Detailed quantitative roughness metrics were reported for each surface. The average roughness (Sa) varied nearly tenfold across the samples, ranging from 0.2 to 2.0 µm, with the machined surface presenting the lowest value and the sandblasted, acid-etched surface showing the highest.

A strong, positive linear correlation was observed between average roughness and interfacial shear strength. However, while roughness varied tenfold, the difference in shear strength was more moderate—about fourfold—suggesting a nonlinear or threshold effect in roughness-induced mechanical enhancement. Crucially, the study identified the developed interfacial area ratio (Sdr) as the principal contributor to shear strength, accounting for approximately 60% of the variation in interfacial strength. In contrast, Sa, the most frequently used roughness parameter in implant research, accounted for only 12% of the strength, highlighting a significant mismatch between commonly used surface descriptors and their actual mechanical relevance.

Further evidence for the mechanical impact of surface topography was provided by a pull test assessing tensile strength between titanium disks and PMMA [103]. When comparing machined and acid-etched surfaces, the acid-etched titanium exhibited a 2.3-fold increase in tensile strength, again reinforcing the conclusion that micro-scale surface features enhance mechanical bonding at the implant interface. These findings underscore that beyond biological contributions, surface roughness independently improves the mechanical anchorage of implants and that topographical parameters beyond Sa—particularly Sdr—are essential to accurately capture this effect (Fig. 4). It should be noted, however, that these experiments rely on the assumption that the bone–implant contact (BIC) is equivalent across the different surface types, which may not always hold true in vivo [34, 85].

### Biomechanical evaluation

Biomechanical anchorage represents the collective outcome of multiple factors: mechanical interlocking between the implant and bone surfaces, the extent and distribution of BIC, the quality and density of surrounding bone, and the interfacial adhesion at the molecular level. Most biomechanical studies evaluating implant osseointegration have focused on comparing two surface topographies—predominantly, machined versus microrough titanium surfaces. A critical distinction should be made when interpreting these results: whether surface topography merely accelerates osseointegration or also enhances its final strength. Acceleration is reflected by improved biomechanical strength at early healing stages, while enhancement implies increased strength at the late stage.

In a rabbit femur model, microrough titanium surfaces featuring 1–2 µm-deep micropits produced a four-fold increase in removal torque at 2 months post-implantation compared to machined surfaces, indicating enhanced osseointegration [104]. A follow-up study using the same model demonstrated a 3.5-fold increase in removal torque at 1 month for the microrough surface, confirming an acceleration of osseointegration [105]. Notably, the strength difference observed at 2 months was sustained through 3 months, suggesting that microrough surfaces not only accelerated but also enhanced the final level of osseointegration.

A similar trend was observed in a rat femur model using the push-in test at healing intervals of 2, 4, and 8 weeks—representing early, mid, and late healing stages [95]. At all time points, push-in values were two- to three-fold higher for microrough implants than machined ones, reinforcing the dual effect of acceleration and enhancement. Interestingly, this study also reported that microrough surfaces retained more mineralized tissue remnants than machined surfaces, implying that the interfacial bond may exceed the intrinsic mechanical strength of bone. This could reflect either stronger mechanical interlocking or topography-induced modulation of interfacial adhesion properties. Consistent findings across other studies have shown that microrough surfaces yield significantly greater biomechanical anchorage than machined surfaces [2, 106].

Studies comparing multiple microrough surfaces also provide valuable insights. A rabbit study examined implants treated with fine (25 µm) and coarse (250 µm) sandblasting, producing micro- and supra-micron-scale roughness, respectively [107]. The average roughness (Sa) was 1.16 µm for the fine-blasted surface and 1.88 µm for the coarse-blasted one, with larger irregularities observed under profilometry in the latter. After 4 weeks of healing, removal torque values were not significantly different between the two surfaces, despite the fine-blasted surface exhibiting 30% greater BIC. This finding highlights the nuanced roles of surface features—while micro-scale roughness may promote more bone formation, supra-micron-scale features may improve mechanical interlocking, offsetting the lower BIC.

A study using a mini-pig model compared implants with microrough surfaces created by acid etching alone to those treated with sandblasting followed by acid etching [108]. The acid-etched-only implants exhibited an Ra of 1.3 µm, while the sandblasted and acid-etched implants reached an Ra of 2.0 µm, reflecting the addition of supra-micron-scale irregularities. Across healing periods of 4, 8, and 12 weeks, the sandblasted and acid-etched surfaces consistently showed 1.7- to 2.3-fold higher removal torque values. Although the implants used in each group differed in shape—warranting cautious interpretation—these findings partially support the concept that while micro-scale topography is critical for stimulating bone formation, the inclusion of supra-micron roughness further enhances mechanical interlocking and contributes substantially to implant anchorage.

As emphasized earlier, it is important to interpret roughness values as relative rather than absolute indicators. Ra or Sa alone does not sufficiently distinguish nano-, micro-, and supra-micron scale features. A comprehensive assessment of biomechanical anchorage requires careful analysis of multiple parameters along with qualitative surface characteristics and functional responses.

### Histological and histomorphometric evaluation

#### Static snapshots of a dynamic process: standard, limitation, and interpretation

Histological and histomorphometric analyses have long served as foundational methods for evaluating bone responses to implant surfaces. These techniques offer direct visualization of bone–implant interfaces, providing valuable insight into tissue morphology, bone contact, and spatial distribution of newly formed bone. However, their interpretative power comes with significant limitations that must be acknowledged when assessing the biological performance of different surface topographies.

Histological evaluation typically relies on cross-sectional images obtained at one or two selected time points. These snapshots represent static outcomes of inherently dynamic processes such as bone remodeling, osseointegration, and largely unknown interfacial adaptation. Moreover, most histological reports present only a single representative section or a small region of the implant surface—often in a single thread area—limiting generalizability and introducing potential sampling bias. As a result, interpretations based solely on histological images can be misleading or overly simplistic when used to compare implant surfaces.

Histomorphometric parameters, such as BIC and peri-implant bone volume, serve as widely accepted quantitative metrics for evaluating osseointegration. However, these measurements are frequently reported without adequate definition or contextual analysis regarding bone quality, remodeling dynamics, or the underlying biological mechanisms. Moreover, such metrics are typically derived from a limited region—often a single thread or the most favorable thread area—raising concerns about representativeness. Given these limitations, the following subsection is organized around key questions aimed at examining the influence of implant surface roughness and topography, while also critically evaluating the significance and constraints of histological findings. These questions include:

(1) Does surface topography affect bone formation around implants?

(2) Are bone volume and BIC correlated? Are BIC, bone volume, and speed of bone formation interrelated?

(3) Is the interfacial strength or the intrinsic mechanical property of osseointegrated bone consistent across different titanium surfaces?

#### Does surface topography affect bone formation around implants?

**Fig. 5:**
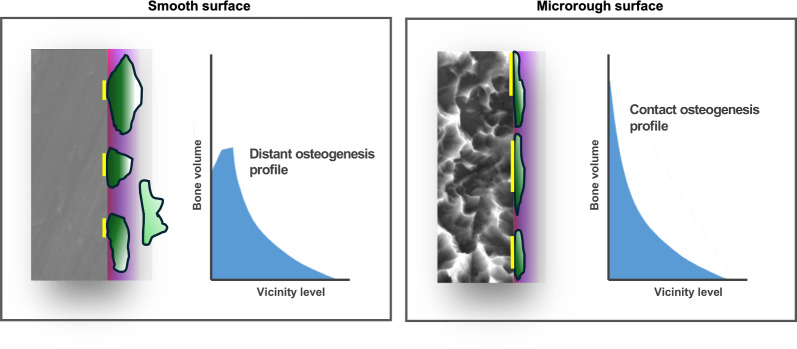
Contrasting bone integration profiles between machined (smooth) and microrough implant surfaces. Bone volume is plotted relative to distance from the implant surface. Around smooth, machined surfaces, bone formation typically shows a dip in volume at the immediate interface, often attributed to soft tissue infiltration. Bone volume recovers only at a distance—an integration pattern known as distant osteogenesis. In contrast, microrough surfaces support direct bone formation at the interface, producing an elevated bone volume adjacent to the surface without an initial dip, despite a relatively lower bone volume farther away. This is defined as contact osteogenesis. As a result, bone–implant contact (BIC; *yellow lines*) is substantially greater on microrough surfaces, whereas overall bone volume at a distance is often higher around smooth surfaces. *Green* represents newly formed bone, *yellow lines* indicate direct BIC, and *red areas* signify soft tissue presence or infiltration

This question has been widely studied, and the answer is generally yes—surface topography does affect peri-implant bone formation. However, the relationship is more complex than it may initially appear due to the unique anatomy and biological dynamics of osseointegration. The osseointegration process involves a time-dependent evolution of bone healing—from an initially broad peri-implant bone formation, to subsequent remodeling and consolidation into a narrower band of bone that persists along the implant surface. This final bone phenotype is anatomically and biologically distinct from the transient bone formed in the osteotomy site without an implant or more distant regions during healing [31, 85]. Because bone formation around implants is a reactive event influenced by the implant surface, its characteristics change depending on proximity to the surface as illustrated in Fig. 5. Therefore, bone morphometry must be carefully analyzed at different time points and spatial zones to yield valid conclusions. In short, osteoconduction—bone formation guided by the implant surface—and osteogenesis—bone formation resulting from the surgical trauma—occur simultaneously but have different triggers.

A substantial body of evidence supports the notion that titanium surfaces with micro-roughness enhance osteoconductivity. Numerous studies comparing smooth surfaces (machined, milled, or polished) with micro-rough surfaces (typically created via acid-etching or similar techniques) have shown that rough surfaces increase early-stage BIC [31]. Bone formation profile is steeper toward the implant interface on microrough surfaces than on microrough surfaces (Fig. 5). Moreover, the degree of BIC is often positively correlated with surface roughness, indicating that rougher surfaces promote more extensive peri-implant bone formation [2, 107, 109, 110].

An important next question is whether there exists a threshold beyond which increased surface roughness no longer enhances bone formation. For example, supra-micron roughness generated by titanium plasma spraying or coarse sandblasting has not consistently shown superior bone formation compared to conventional microrough surfaces [107, 111]. It is also noteworthy that, even under optimal conditions and with analyses focused on the “best” regions, most reported BIC values fall within the 40–60% range, with values exceeding 70% being exceptionally rare [87, 99]. Furthermore, titanium surfaces with substantially greater micro-level roughness than current microrough standards have yet to be developed, either commercially or experimentally, making it challenging to definitively address this threshold question.

Further questions arise as to whether the increase in BIC is due to an actual increase in bone volume, a broader extent of bone formation, a reduced incidence of soft tissue intervention at the interface, or a combination of these factors. These possibilities will be addressed in the next section.

#### Are bone volume and BIC correlated? Are BIC, bone volume, and speed of bone formation interrelated?

A key study emphasized the importance of evaluating de novo bone formation—bone newly generated in response to the implant—rather than assessing all bone tissue present around the implant [31]. As previously discussed, bone formation in the peri-implant region results from two distinct biological processes: (1) the remodeling or preservation of pre-existing bone, including surgical-reactive bone, and (2) the formation of new, osseointegrating bone. The former is primarily influenced by the native anatomical environment and the body's generalized wound-healing response to surgical trauma, largely independent of the implant itself. In contrast, de novo osseointegration represents a distinct and targeted biological response, initiated by and directed toward the implant surface.

To isolate and evaluate implant-induced bone formation, researchers developed a specialized experimental implant for rat femurs featuring a rectangular chamber that allowed ingrowth exclusively of newly formed bone [31]. By examining histological cross-sections of this chamber, the analysis excluded innate bone or surgically induced healing, allowing a focus solely on de novo osteogenesis. When comparing machined implants to those with acid-etched micro-rough surfaces, they found that bone around microrough surfaces was thinner but more continuous and extensive, whereas bone around machined implants was thicker yet fragmented. As a result, total bone volume was either slightly higher around machined surfaces or comparable between the two, depending on the healing duration. A particularly noteworthy finding was the frequent presence of soft tissue interposition at the implant interface in the machined group, leading to significantly lower BIC. In contrast, microrough implants consistently showed greater BIC across both early and late healing phases. These results underscore the distinct modes of bone formation: contact osteogenesis predominates on microrough surfaces, while distant osteogenesis is more typical of smoother surfaces. However, it is important to note that microrough surfaces did not increase the total volume of bone formed. These contrasting bone formation patterns are illustrated in Fig. 5.

Regarding the speed of osseointegration, the study provided additional insights by separately analyzing bone formation in the near zone (within 50 µm of the implant surface) and the far zone [31]. At week 2 of healing, bone volume in the near zone was three times higher around microrough surfaces than around machined ones, suggesting accelerated bone formation relevant to osseointegration. Interestingly, by week 4, bone volume in the near zone was higher for machined implants, although the difference was not statistically significant. In the far zone, bone volume showed no significant difference between the two implant types at both time points.

These findings suggest that peri-implant bone volume and the rate of bone formation can exhibit a positive correlation under specific analytical conditions and time points. However, this relationship may be absent—or even inverted—under different circumstances. Such inverse correlations are biologically plausible when considering the inherent trade-off faced by osteoblasts, as discussed earlier and illustrated in Fig. 2. We hypothesize that this reverse correlation is less frequently observed in vivo due to the continuous and abundant recruitment of osteogenic cells from the host environment, in contrast to the single-time cell seeding used in in vitro systems. Nevertheless, a general trend persists: although microrough surfaces consistently accelerate early bone formation compared to machined or smooth surfaces, the resulting bone tends to be thinner and does not significantly increase the total bone volume.

A systematic review of 14 in vivo studies reported a positive correlation between titanium surface roughness and both BIC and mechanical push-out strength [112]. Another review highlighted the particular efficacy of microrough surfaces in host sites with low bone quality [113]. However, neither review addressed the relationship between the rate of osseointegration and BIC, underscoring the difficulty of evaluating temporal dynamics using conventional cross-sectional analyses.

#### Reimagining osseointegration: is the intrinsic mechanical property of osseointegrated bone and interfacial adhesion consistent across different titanium surfaces?

BIC and the mechanical stability of osseointegration are often treated as primary indicators of implant success. However, these surface-level metrics—though essential—may not fully capture the biological and mechanical complexity underlying osseointegration. Two implants may present similar BIC values, yet the intrinsic quality of the bone tissue involved and the strength of the bone–implant interface can differ dramatically. To delve deeper into these distinctions, a series of in vitro studies examined the mechanical properties of mineralized matrix formed on titanium surfaces with different micro-topographies—specifically, comparing machined (smooth) versus acid-etched microrough surfaces [39, 60, 62]. Using nano-indentation analysis, researchers assessed the hardness and elastic modulus of bone matrix synthesized over 28 days of culture. The results were striking: mineralized matrix developed on microrough surfaces was approximately 3 to 3.5 times harder and 2.5 to 3 times stiffer than that formed on machined surfaces [39]. These differences were not merely quantitative—they were biologically meaningful, mirroring the contrast seen between cortical and trabecular bone in terms of mechanical competence. Enhanced hardness and stiffness were also associated with increased calcification and higher collagen density, suggesting that microrough surfaces stimulate faster osteoblastic differentiation but also promote the production of higher-quality matrix.

Crucially, these findings were corroborated in vivo. Bone tissue formed around microrough titanium implants exhibited mechanical hardness values that were three times greater than those formed around machined implants, both at the immediate bone–implant interface and in the surrounding peri-implant bone [58]. Notably, the hardness of bone integrated with microrough implants approached that of native cortical bone, whereas bone adjacent to machined implants resembled trabecular bone in its mechanical properties. These results underscore the ability of surface microtopography to enhance not only the biological integration but also the mechanical integrity of osseointegrated bone.

Contact or adhesion—long an overlooked question at the titanium–bone interface—has emerged as a critical determinant of osseointegration. Beyond the inherent mechanical properties of bone, the interfacial strength between the mineralized matrix and the titanium surface plays a pivotal role. This raises the hypothesis: does a molecular “glue” exist at the titanium–bone interface, and if so, can it be modulated by surface roughness or topography? To address this, a series of studies employed nano-scratch testing to quantify the adhesive strength between mineralized matrix and titanium surfaces [60, 62, 114]. After 24 days of osteogenic culture, the critical load required to delaminate the bone matrix from microrough surfaces was found to be approximately 70% higher than that required for machined surfaces [114]. This interfacial reinforcement was biologically linked to the upregulation of genes involved in the synthesis of proteoglycans and glycosaminoglycans (GAGs)—key components of the bone extracellular matrix known to facilitate adhesion.

The functional role of these molecules was further confirmed by enzymatic experiments: when glycosaminoglycan-degrading enzymes were added to the cultures, the interfacial strength between bone matrix and titanium surface dropped significantly [61, 114]. These findings indicate that microrough surfaces not only modulate the behavior of osteoblasts to produce mechanically superior matrix but also create a more biologically adhesive interface through enhanced matrix composition.

Taken together, this body of evidence challenges the conventional notion that BIC or push-out strength alone can define the success of osseointegration. The surface characteristics of titanium implants profoundly influence both the quality of the bone that integrates and the strength of the interface where this integration occurs. Microrough surfaces, in particular, appear to facilitate a synergistic enhancement of both the intrinsic mechanical properties of osseointegrated bone and the biological integrity of the bone–implant interface. Reimagining osseointegration through this more nuanced lens may open new avenues for optimizing implant design and long-term clinical outcomes.

### Creating dichotomy of osteoblasts: cellular response to surface roughness and topography

An analytical experiment varying titanium surface roughness provided compelling evidence for the dichotomous kinetic behavior of osteoblasts in response to surface topography [98]. Using a standardized model of rat bone marrow-derived osteoblasts cultured on titanium disks with four distinct surface finishes—machined, sandblasted, acid-etched, and sandblasted + acid-etched—they observed opposing trends in two critical biological responses: initial cell recruitment and subsequent cell retention. After 24 h of incubation followed by a controlled detachment procedure using vibration and trypsinization, surfaces with higher roughness—particularly the sandblasted + acid-etched specimens—demonstrated significantly enhanced retention of osteoblasts. In contrast, those same rougher surfaces recruited fewer cells within the same incubation period, indicating that increased topographical complexity may present a barrier to initial cell attachment while concurrently promoting stronger anchorage for cells that do succeed in attaching.

This inverse relationship was strongly tied to quantifiable surface parameters. Among the 3D roughness metrics evaluated, Sdr (developed interfacial area ratio) emerged as the most predictive determinant for both osteoblast recruitment and retention (Fig. 4). Higher Sdr values correlated linearly with stronger cell retention capacity, but inversely with cell recruitment numbers. The average roughness (Sa) also showed meaningful correlations, though less consistently than Sdr. These findings underscore the importance of incorporating multidimensional surface metrics—particularly those reflecting surface area expansion and spatial complexity—when assessing or designing implant surfaces for biological performance. The dichotomous behavior observed supports the hypothesis that surface roughness induces functional divergence in osteoblastic responses, suggesting that optimizing implant surfaces requires a nuanced balance between different cellular events.

We next explore in detail the inverse correlation between the volume and speed of osseointegration. Broadly speaking, the number of cells—determined by the rate of proliferation—governs the quantity of bone formed [115], while the rate of differentiation dictates the speed at which bone formation occurs. One of the most consistently observed effects of increasing surface roughness is a significant suppression of cell proliferation. Osteoblast-like cells and mesenchymal stem cells proliferate at markedly lower rates on microrough surfaces compared to machined or polished titanium. For example, BrdU incorporation assays have revealed that cell proliferation on acid-etched surfaces is approximately 50% lower than on machined surfaces at early and mid-time points of culture [116]. Longitudinal studies confirm that this trend persists, with proliferation rates on smooth surfaces exceeding those on roughened surfaces by as much as 40% even after one week of culture [117]. Surface-dependent variations in proliferation were also evident across a range of roughened substrates: titanium plasma-sprayed (TPS) surfaces, the roughest among those tested, supported only one-third the proliferation seen on electropolished titanium after 24 h [118]. These findings support the general consensus that increased surface roughness leads to a reduction in the proliferation of osteoprogenitor cells [34, 36, 38, 39, 119, 120].

Paradoxically, while cell proliferation is downregulated on rough surfaces, osteogenic differentiation is robustly promoted. Numerous studies have demonstrated that microrough surfaces upregulate osteoblast-specific gene and protein markers such as alkaline phosphatase (ALP), collagen type I, bone sialoprotein, osteocalcin, and osteopontin [96]. For instance, sandblasted titanium surfaces induced significantly higher osteocalcin expression than machined surfaces when stimulated with active vitamin D3 [121]. Similarly, ALP activity is consistently elevated on sandpaper-roughened titanium, and grit-blasted titanium alloys increase the expression of transforming growth factor-β1 and osteoprotegerin [122]. These findings are further supported by multiple reports demonstrating enhanced osteoblastic differentiation on roughened titanium surfaces [50, 94, 118, 123], contributing to the accelerated and more robust BIC often observed in vivo with such surface modifications.

This phenomenon represents a biological dichotomy—or more precisely, a kinetic dilemma—of osteoblasts [37, 123, 124]. The same surface roughness that favors differentiation simultaneously suppresses proliferation. This inverse relationship is well recognized in bone biology and is often attributed to molecular switches that regulate the cell cycle in favor of lineage commitment. For example, cyclin D1 and proliferating cell nuclear antigen (PCNA), whose co-expression is associated with cell cycle depression, is upregulated on rough surfaces [37]. In contrast, transcription factors like Runx2 are upregulated and act as gatekeepers to initiate osteogenic differentiation, effectively guiding cells to exit the proliferative phase [125]. This trade-off suggests that rough surfaces push cells to commit to bone formation earlier, at the expense of self-renewal or expansion.

Curiously, this dichotomy—so prominent in vitro—is rarely observed in vivo across the literature. A plausible explanation lies in the fundamental difference in cellular dynamics. In vitro models typically depend on a one-time seeding of a limited number of cells, which are then subject to modulation by the surface properties of the material. In contrast, the in vivo environment provides a continuous and dynamic influx of osteogenic precursor cells via blood vessels and bone marrow, ensuring a consistent cellular supply to colonize the implant surface. As a result, although microrough surfaces may suppress proliferation on a per-cell basis, this effect may be masked by the ongoing recruitment of new cells, allowing osseointegration to proceed without a marked reduction in bone volume—or possibly causing such reductions to go unnoticed. Nevertheless, as discussed earlier, thinner bone formation around microrough surfaces has been documented in several well-controlled histological studies, indicating that this limitation is real and detectable under appropriate analytical conditions. More importantly, BIC never reaches 100%, underscoring a critical limitation in cellular proliferation on roughened implant surfaces. It is therefore essential not to assume that rougher surfaces are inherently superior to smoother ones in every facet of osseointegration.

This understanding opens a new frontier in implant surface design. If future surface technologies can overcome or mitigate the intrinsic osteoblast dichotomy—whether by decoupling the two processes, sequentially stimulating them, or compensating for one while preserving the other—we could achieve more efficient osseointegration in both speed and quantity. Implant surfaces that simultaneously support early cell expansion and timely differentiation would represent a new generation of biomaterials, capable of accelerating healing and improving long-term implant anchorage.

### Effect of nanotopography/nanoroughness

Following the biological success and technical maturation of microrough surfaces, the field has turned its attention to nanotopography as the next frontier in implant surface technology. The strategy behind this shift is twofold: to introduce novel biological functions not achievable with microscale textures, and to overcome known limitations of microrough surfaces, particularly their suppression of osteoblast proliferation [36]. Nanostructured surfaces are expected to facilitate early cell attachment and expansion while maintaining or enhancing differentiation, potentially accelerating osseointegration and improving healing outcomes.

The primary challenge in developing nanostructured titanium surfaces lies in the ability to create defined, reproducible, and biologically effective nanomorphologies. While experimental techniques such as self-assembly, modified acid etching, and chemical deposition have been used to create controlled nanonodules or other polymorphic nanostructures [69, 90–92, 126–128], few have successfully translated into commercial dental implants [36]. Additionally, a clear distinction must be made between topographical and chemical modifications, as many so-called nanofeatured surfaces derive their nanoscale features from non-titanium materials such as calcium phosphate deposition or coating [129].

Several experimental studies have addressed this challenge by designing hybrid micro-nano titanium surfaces, where well-controlled nanonodules are superimposed onto conventional microrough textures [127, 130]. For instance, titanium surfaces with 300-nm nanonodules formed by controlled self-assembly significantly enhanced osteoblast attachment, proliferation, and differentiation compared to microrough surfaces alone. These hybrid surfaces were also superior to smooth surfaces in supporting early cell recruitment and proliferation. In vivo biomechanical testing further confirmed that these implants achieved more than threefold stronger bone integration than standard microrough implants. Interestingly, the hybrid surfaces selectively suppressed fibroblast growth while promoting osteoblast function, offering additional biological advantages [38]. A recent review comprehensively summarized the underlying mechanisms and ongoing challenges associated with nano-surface technology in the field of implantology [35].

Among commercially available implants, only a few incorporate nanostructures directly on titanium surfaces [35]. OsseoSpeed® (Astra) is a microrough implant modified with fluoride to produce undefined nanonodular features. These nanostructures may accelerate early osseointegration but do not appear to improve final bone integration outcomes over its microrough predecessor. Other products, such as SLActive® (Straumann) and NanoTite™ (Zimmer Biomet), include nanoscale features, but their origins lie in salt crystallization or deposition of calcium phosphate, respectively. Because their nanofeatures are chemically derived and not topographically or structurally defined on titanium itself, they fall outside the scope of this review.

In summary, while commercial applications of true nanostructured titanium surfaces remain limited, controlled experimental models demonstrate promising potential for micro-nano hybrid surfaces to resolve the biological trade-offs associated with rough surfaces. Future developments should focus on precision-engineered nanotopography that can be reliably fabricated on titanium and incorporated into manufacturing workflows, opening a pathway to the next generation of implant surfaces.

### Critical insights and impending challenges in surface technology and science

Despite decades of dedicated research and a wealth of data on the role of implant surface modification in promoting osseointegration, surface science in implantology stands at a critical juncture. While microrough surfaces have become the cornerstone of contemporary implant design, the field’s next advancement is impeded by several overlooked yet fundamental limitations in both experimental design and surface engineering strategy.

The first and most striking issue is the lack of comparative understanding among microrough surfaces themselves. Historically, studies have overwhelmingly relied on a binary comparison—contrasting a single microrough surface with a machined control. This oversimplified approach has generated broad conclusions about “roughness,” but left unanswered which microrough surface performs best, under which conditions, and why. Without rigorous, side-by-side evaluation among diverse microrough surfaces, meaningful differentiation, optimization, and selection for specific clinical scenarios remains impossible.

Second, the field has narrowly focused on average roughness values—such as Sa—as the defining metric, ignoring the rich topographical diversity that rough surfaces embody. Two surfaces can share the same Sa yet exhibit profoundly different biological responses due to differences in their morphological traits: random vs. ordered, uniform vs. heterogeneous, sharp vs. rounded features, or rough vs. porous micro-architecture. These topographical nuances are likely as influential—if not more so—than roughness magnitude alone, and they remain largely uncharacterized and underexploited in implant design. We must reframe roughness not as a scalar value, but as a structural language that cells interpret in context.

Third—and perhaps most fundamentally—current microrough surfaces are not the result of deliberate engineering but rather incidental byproducts of fabrication processes. There is no standardized or precisely controllable method to produce surfaces with incrementally varied roughness while preserving consistent topographical characteristics. Consequently, acid-etched microrough surfaces are not truly designed; they are discovered. This lack of intentionality renders biological outcomes unpredictable until empirically tested, impeding progress and limiting the ability to rationally target specific biological responses—such as addressing the kinetic dichotomy of osteoblast behavior, wherein proliferation and differentiation are inversely influenced by surface roughness. In the absence of engineering control, surface optimization remains a matter of trial and error rather than strategic design.

The implications of this lack of precision are significant. There is no established correlation curve linking roughness levels with osteogenic outcomes, nor has an upper threshold—or “ceiling”—for surface roughness efficacy been defined. We simply do not know whether further increasing roughness would produce additional biological benefits, because no surface has been purposefully designed to test this hypothesis in a systematic manner. Indeed, the development of microrough surfaces appears to have plateaued, with most designs converging around an Sa value between 1.0 and 2.0 µm. Moreover, the meso-scale domain (10–100 µm)—despite its critical role in mechanical interlocking and its potential to substantially increase surface area—remains largely unexplored.

To overcome these barriers, future efforts must prioritize the development of rational, controllable surface design technologies. Such a platform would enable systematic creation of surfaces with graded roughness and precisely tuned topographical features—opening the door to predictive biology, where the impact of a surface can be anticipated, not just observed. This would finally allow the field to move beyond empirical iteration and into the realm of strategic surface engineering, capable of addressing unresolved biological challenges and unlocking the next generation of implant performance.

### Summary of dimension 2: topography/roughness

Surface topography is undoubtedly a central determinant of implant osseointegration. Microrough surfaces, typically produced by acid-etching, have revolutionized implant dentistry by promoting osteogenic differentiation and accelerating bone–implant integration. However, this success is accompanied by a trade-off—while rough surfaces enhance differentiation, they tend to suppress cell proliferation, creating a biological dichotomy that has yet to be fully addressed.

In pursuit of further enhancement, nanotechnology has been explored as a means to complement microroughness, aiming to preserve differentiation while recovering proliferation. Experimental micro–nano hybrid surfaces, particularly those with defined nanonodular features, have shown promise in overcoming this limitation, significantly boosting osteoblast attachment and proliferation. Nonetheless, among commercially available implants, nanofeatures are either chemically derived or undefined, and true nanostructured titanium surfaces remain rare and under-characterized.

Despite decades of innovation, current surface technologies face fundamental limitations. Comparative data among various microrough surfaces is scarce, and the field lacks systematic models to isolate and study the role of individual topographic traits. Critical morphological parameters beyond average roughness—such as texture regularity, porosity, and scale—remain underexplored. Furthermore, meso-scale topography (10–100 µm), which could greatly enhance mechanical interlocking and surface area, is largely neglected.

This chapter highlights the urgent need for a new generation of engineered surfaces: ones that are precisely designed, biologically informed, and strategically tailored to maximize both mechanical and cellular outcomes. The path forward requires controlled and comparative approaches to surface design, enabling a shift from descriptive science to predictive and functional biomaterial engineering.

## Dimension 3—time: the missing variable in osseointegration

### Background: chemical inertness and biological passivity—is it proven or unchallenged?

Among various biomaterials and implant materials, titanium has long been praised for its chemical stability and biocompatibility, grounded in the belief that its surface is chemically inert and thus inherently safe for biological applications (Fig. 7). This inertness arises from the naturally forming titanium dioxide (TiO_2_) layer, typically 2–100 nm thick [99], which provides corrosion resistance and structural integrity due to its strong Ti=O double bonds. The material’s oxide layer is considered both physically and chemically stable, a property attributed to the ideal pairing of titanium’s and oxygen’s valence electrons. However, this notion of chemical inertness, while advantageous for durability, raises a deeper and often overlooked concern: does chemical inertness equate to biological passivity? In other words, does the very stability that protects titanium surfaces also suppress their biological potential?

One clue lies in the surface energy of titanium, which significantly declines after manufacturing. Clean titanium surfaces, when freshly prepared, are highly hydrophilic and bioactive [13, 131–133]. Yet, within minutes to hours—even under clean conditions—they begin to adsorb atmospheric hydrocarbons and organic contaminants [9–11]. This contamination diminishes surface energy, alters physicochemical interactions, and profoundly suppresses cellular responses. The phenomenon, now known as the biological aging of titanium [9, 10, 134, 135], challenges the long-held assumption that the bioactivity of titanium surfaces is permanent and unchanging.

This shift in understanding has crucial implications for three unresolved questions in implant science. First, why does bone–implant contact (BIC) plateau at suboptimal levels—typically around 45–75% [31, 136–138]—even under ideal biological conditions and extended healing durations? In environments such as bone marrow, where stem cells and osteogenic potential are abundant, full osseointegration should theoretically be achievable. Second, why do some implants fail with unknown reason, despite appropriately placed? Conventional explanations, such as limited stem cell supply, remain hypothetical and insufficient. Third, why do in vitro and in vivo experimental outcomes in literature often conflict, even when using similar implant surfaces and study models?

These longstanding questions may share a common root: the unrecognized and unaddressed role of time. Titanium surfaces do not remain biologically static after manufacturing—they undergo temporal degradation in bioactivity. Yet, implant surface technologies have not accounted for this dynamic property, and biological time has not been treated as a variable in the design, storage, or experimental and clinical application of implants. This chapter explores time as the third and missing dimension in titanium surface science. By recognizing and addressing the time-dependent decline in surface bioactivity, we may not only answer unresolved questions but also uncover new strategies to restore, preserve, or even enhance the biological performance of titanium implants throughout their lifecycle.

### Discovery of biological aging: hydrophilicity loss, carbon accumulation, and electrostatic potential as as critical temporal dynamics of physicochemical properties

Among the surface characteristics outlined in Fig. 1, physicochemical properties refer to material attributes governed by both physical and chemical principles—such as surface energy, wettability, surface charge, and elemental composition. These properties are distinct from overt chemical modifications, which typically involve deliberate surface alterations like hydroxyapatite coatings or anodic oxidation to incorporate new chemical functionalities into the bulk or surface layer. In contrast, physicochemical properties emerge from the inherent characteristics of the outermost molecular layers—often within a few nanometers—such as the titanium dioxide (TiO_2_) film naturally formed on titanium implants. Though subtle, these surface-level properties play a critical role in dictating how a material interacts with its biological environment.

One of the most transformative insights in recent implant science is the concept of biological aging—a time-dependent decline in the surface bioactivity of titanium caused by progressive alterations in its physicochemical state. This aging process is primarily characterized by three interrelated phenomena: (1) the gradual loss of surface hydrophilicity, (2) the accumulation of hydrocarbon contaminants, and (3) a shift in electrostatic surface potential. Notably, these detrimental changes occur without any visible or structural alterations to the implant, yet they significantly compromise its biological performance. Understanding and mitigating these temporal dynamics is essential for maintaining the bioactivity of titanium surfaces from manufacturing through clinical application.

#### Hydrophilicity loss

The first recognized manifestation of biological aging was the gradual loss of hydrophilicity on titanium surfaces over time [9, 134, 139, 140]. A freshly prepared acid-etched titanium surface—within minutes of preparation—displays superhydrophilicity, defined as a water contact angle less than 5°, indicating high surface energy and strong affinity to aqueous environments. As the surface ages, however, this property degrades progressively: 3-day- and 7-day-old titanium surfaces remain moderately hydrophilic, while a 28-day-old surface typically becomes hydrophobic—with water contact angles exceeding 65°. By 3 months, the water contact angle of a commonly produced acid-etched surface often reaches 80° or higher, reflecting a complete reversal in surface wettability.

This change occurs under standard storage conditions—ambient air, room temperature, and darkness—indicating that even in clean environments, titanium surfaces degrade in their reactivity. Commercially available implants, stored and shipped for months after production, thus present to the surgeon not as hydrophilic surfaces, but as hydrophobic ones[4, 12, 18, 131, 141–144].

#### Carbon accumulation

Closely linked to the loss of hydrophilicity is the accumulation of carbon-based contaminants on the titanium surface (Fig. 1). Atmospheric hydrocarbons—organic molecules made of hydrogen and carbon (e.g., C_x_H_γ_)—are present in ambient air at concentrations of around 3 parts per million. These invisible, odorless molecules are highly mobile and adsorb readily onto reactive surfaces such as freshly prepared titanium. This carbon contamination begins almost immediately after surface preparation and increases with time [9, 10].

In longitudinal studies, the atomic percentage of surface carbon rises from approximately 15% on a fresh titanium surface to 60% on a 4-week-old surface. Concomitantly, the atomic percentages of titanium and oxygen drop from 25 to 10% and 75 to 30%, respectively—suggesting that aged titanium surfaces are no longer predominantly titanium dioxide, but instead become dominated by organic carbon compounds. Notably, studies have found that commercial implants routinely carry carbon loads of 40–75% on their surfaces [9, 10]. This contamination directly contributes to the loss of surface energy and hydrophilicity loss. The contaminated carbon layer has been defined as carbon pellicle [13, 132, 145–147].

#### Electrostatic surface potential

A third, emerging physicochemical parameter implicated in biological aging is the electrostatic surface potential of titanium. Traditionally, titanium has been considered a bioinert material, largely due to its net negative surface charge under physiological conditions [148–150]. While this has been regarded as a benign property, it poses a fundamental biological challenge: many key proteins involved in osseointegration—such as fibronectin, osteopontin, and various integrins—also carry a net negative charge at physiological pH [151]. The resulting electrostatic repulsion between the negatively charged implant surface and these proteins can hinder their initial adsorption, compromising subsequent cell adhesion and signaling. Moreover, cell membranes themselves are negatively charged, further exacerbating the repulsive interaction between the titanium surface and incoming cells. This electrostatic incompatibility may contribute to the reduced biological performance of aged titanium surfaces, emphasizing the importance of surface charge modulation as a potential strategy for improving osseointegration.

To overcome this, surface conditioning with divalent cations, such as Ca^2^⁺ and Mg^2^⁺, are required to deploy to form ionic bridges between the negatively charged titanium surface and anionic proteins or cells, as assumed to occur in physiological environment [148–150]. In contrast, monovalent cations like Na⁺ or K⁺ fail to support this bridging effect. However, studies suggest that newly prepared titanium surfaces exhibit electropositive characteristics, capable of attracting anionic proteins without the need for divalent cations [9, 134]. This implies that the surface charge polarity of titanium shifts from positive (in fresh state) to negative (as it ages), although this phenomenon remains hypothetical and requires further investigation.

Collectively, these time-dependent changes in hydrophilicity, carbon contamination, and surface charge represent a fundamental shift in how titanium implants should be understood. Rather than being chemically inert and biologically stable, titanium surfaces are dynamic, temporally sensitive systems whose reactivity degrades predictably over time. This has far-reaching implications for implant design, storage, and clinical performance—necessitating new strategies to preserve or restore surface bioactivity at the point of implantation.

### Interrelation of the three physicochemical properties

The three key physicochemical properties discussed—hydrophilicity loss, carbon accumulation, and electrostatic potential shift—are not isolated phenomena but instead appear to be interconnected, with overlapping mechanistic bases and temporal co-evolution during the biological aging of titanium.

A critical connection exists between carbon accumulation and surface charge. Hydrocarbon molecules, which constitute atmospheric organic contaminants, are composed primarily of carbon and hydrogen atoms (CxHy) [13]. The electronegativity difference between carbon and hydrogen is relatively small (ΔEN ≈ 0.3), resulting in weak dipole moments and rendering hydrocarbons largely nonpolar or only slightly electronegative. However, the net deposition of hydrocarbon molecules onto titanium surfaces introduces a subtle electronegative character, which may shift the overall surface polarity of titanium toward the negative side. This aligns with the historical understanding that aged titanium surfaces repel negatively charged biomolecules, such as fibronectin and certain cell membranes, unless bridged by divalent cations like Ca^2^⁺ or Mg^2^⁺. In contrast, freshly prepared titanium surfaces appear to exhibit electropositive character, facilitating spontaneous adsorption of anionic biomolecules without ionic mediation. This polarity reversal is thus at least partly attributable to the increasing carbon load on the surface over time.

Similarly, a strong mechanistic link is observed between carbon accumulation and hydrophilicity loss [9]. Water is a highly polar molecule, and its interaction with other materials depends heavily on the polarity and surface energy of those materials. Surfaces rich in polar functional groups (e.g., hydroxyls or oxides) tend to attract water and become hydrophilic. On the other hand, hydrocarbons are fundamentally nonpolar, and their accumulation introduces a low-energy, nonpolar layer on the titanium surface [152]. This nonpolar coating repels water molecules, diminishing the surface's wettability and converting a previously hydrophilic surface into a hydrophobic one. This explains the temporal degradation of hydrophilicity, where new titanium exhibits superhydrophilicity, but aged titanium becomes increasingly hydrophobic as carbon content rises.

An additional hypothesis is that newly prepared titanium surfaces themselves are intrinsically polar, possibly due to surface hydroxylation or the structural characteristics of titanium dioxide. The rapid spreading of water droplets observed on freshly prepared titanium supports this idea. However, further studies are needed to directly measure surface dipole moments or the nature of exposed chemical groups on new titanium. Regardless, what is well established is that the loss of polarity over time correlates with both carbon accumulation and hydrophilicity loss, strongly suggesting a causal cascade initiated by surface hydrocarbon deposition.

In this context, carbonization—or the accumulation of a carbon pellicle—can be regarded as the primary physicochemical event driving the biological aging of titanium surfaces [9, 13, 135]. The subsequent loss of hydrophilicity and alteration in electrostatic potential appear to be secondary consequences of this surface contamination. One practical advantage of assessing hydrophilicity is its visual detectability, offering a straightforward indication of biological aging. In contrast, changes in electrostatic potential and elemental composition require specialized instrumentation and complex analyses to detect. These findings prompt a critical biological question: Do these time-dependent physicochemical changes compromise the biocompatibility and osseointegrative capacity of titanium implants? As explored in the following sections, accumulating evidence suggests that they do—substantially.

### Biological evidence: impact of biological aging or the factor of time

To determine whether the time-dependent physicochemical changes described above influence the osteoconductive capacity of titanium—that is, its ability to support bone integration—a series of in vitro and in vivo experiments were conducted using titanium surfaces of varying ages [9]. The experimental groups included freshly acid-etched titanium, as well as titanium aged for 3 days, 2 weeks, and 4 weeks under ambient, dark conditions. In vitro, primary osteoblasts isolated from rat femurs were cultured on these surfaces. A clear negative correlation was observed between titanium age and biological performance. The number of osteoblasts that initially attached to the surface was significantly reduced as titanium aged. On 4-week-old titanium, the number of adherent cells was approximately half that on fresh titanium. Similarly, osteoblastic proliferation was substantially lower on aged surfaces.

Functionally, aged titanium surfaces demonstrated decreased osteoblast activity. Markers such as alkaline phosphatase (ALP) activity and matrix calcium deposition were also reduced by approximately 50% or more on 4-week-old titanium, compared to freshly prepared surfaces. Interestingly, gene expression analysis of osteoblastic differentiation did not reveal significant differences between groups, suggesting that the functional deficits were not due to impaired differentiation, but rather to a lower number of cells adhering and expanding on aged titanium.

The in vivo performance of aged titanium was also markedly impaired. Using a rat femur implant model, the strength of bone-to-implant integration—measured by biomechanical push-in testing—was 37 N for freshly prepared titanium implants, but dropped to 16 N for 4-week-old implants. Histological analysis further revealed that the 4-week-old implants were surrounded by fragmented, discontinuous bone formation, with areas of soft tissue encapsulation evident on parts of the implant surface. In contrast, freshly prepared implants showed robust, contiguous bone formation with minimal to no soft tissue interference. Fresh titanium achieved over 90% BIC, a level rarely matched by aged implants. In contrast, 4-week-old implants reached only 58% BIC. This is consistent with literature reports, which commonly show BIC values for aged or standard titanium implants ranging only from 45 to 65%. These BIC values were recorded after a 4-week healing period, representing the late stage of bone healing in rats, suggesting that the deficit in integration associated with titanium aging is not merely a delay in healing, but rather a permanent reduction in biological performance. Furthermore, a longer-term study tracking implants aged for up to six months revealed continued biological degradation, including diminished recruitment of osteogenic cells and reduced ALP activity [153]. Notably, while hydrophilicity loss saturated at a contact angle of 95° after one month, biological aging continued beyond this point, implying that the surface contamination process—particularly hydrocarbon accumulation—persists and impacts implant performance well beyond the initial loss of hydrophilicity.

To investigate how implant surface properties influence the initial biological response, particularly blood and protein dynamics, a series of computational fluid dynamics (CFD) models were constructed using screw-shaped implants with either hydrophobic (contact angle 70°) or superhydrophilic (contact angle 0°) surfaces [154]. CFD simulations enabled visualization and quantification of blood flow patterns and fibrinogen distribution under constant blood inflow conditions [155, 156]. Remarkably, hydrophobic implants exhibited substantial biological voids, with 40–50% of the implant interface and thread spaces left unoccupied by plasma [154]. In contrast, superhydrophilic surfaces eliminated these voids, ensuring near-complete and rapid blood coverage. Vector analysis of blood flow within threads showed disorganized, outward-moving vectors for hydrophobic implants, while superhydrophilic implants generated vortex nodes and inward-directed vectors, actively pulling blood into the threads. Most notably, fibrinogen accumulation at the superhydrophilic interface was up to 20 times greater than at the hydrophobic surface, indicating dramatically enhanced protein recruitment immediately after implantation. The enhanced blood flow around hydrophilic implants was defined as contact hemodynamics as opposed to distant dynamics around standard, hydrophobic implants.

In a complementary study, the effects of surface topography and wettability were examined using the same CFD approach, focusing on protein recruitment/retention efficiency [157]. While microrough surfaces on their own slowed blood flow by four-fold and limited protein infiltration compared to smooth surfaces, they ultimately doubled the protein recruitment/retention index due to enhanced local retention. However, converting implant surfaces from hydrophobic to superhydrophilic consistently increased protein infiltration by 2–3 times and further slowed blood flow, enhancing protein accumulation in the peri-implant region. The highest efficiency of protein recruitment and retention was achieved with the combination of microrough topography and superhydrophilicity. These findings emphasize that while microtopography contributes to both positive and negative modulation of protein dynamics, superhydrophilicity has a uniformly beneficial effect, greatly improving the early biological environment around implants and potentially facilitating superior osseointegration outcomes.

In summary, a comprehensive set of in vitro and in vivo studies has demonstrated that titanium undergoes a biological aging process marked by a time-dependent decline in bioactivity. This aging converts titanium surfaces from bioactive to bioinert, significantly reducing their ability to support osteoblast attachment, proliferation, and function. The negative impact of aging is especially prominent during the early healing phase but remains consequential even in later stages, suggesting a potentially irreversible loss of biological integration potential due to the passage of time. More recently, evidence is emerging that biological aging of titanium also compromises its ability to recruit and retain blood and wound-healing proteins, revealing an additional, previously unrecognized dimension of functional decline.

### Biological aging in other implant surfaces and materials

While acid-etched microrough surfaces are the most commonly used in dental implants, a variety of other surface topographies—produced through different surface treatments—are also available on the market. Using the same experimental model comparing fresh and 4-week-old surfaces, biological aging has been confirmed on other titanium surface types, including machined and sandblasted surfaces [8, 9], supporting the generalization of this phenomenon across titanium implants. Furthermore, time-dependent degradation of bioactivity has also been demonstrated in other implant materials, with direct evidence observed in cobalt-chromium alloys [8], and indirect evidence noted in titanium alloys [120] and zirconia [73].

### Clinical implications of titanium’s biological aging: time as a hidden determinant of implant success

The biological aging of titanium surfaces introduces a crucial, yet historically overlooked, variable in implant therapy—time. Implants are not used immediately after manufacturing; rather, they undergo transportation, inventory storage, and clinical shelf time, all of which contribute to a progressive loss of surface bioactivity. Traditionally, implant osteoconductivity has been assumed to be stable, and products are marketed as storable medical devices with little consideration of biological degradation. However, as the studies above clearly demonstrate, titanium’s ability to support osseointegration declines significantly with time, suggesting that many commercially available implants may have already lost a substantial portion of their biological potential before clinical use.

This new understanding presents a critical opportunity in patient care. The phenomenon of biological aging can be harnessed in two strategic directions: prevention and restoration. By preventing surface degradation through optimized packaging, storage, or surface stabilization, or by restoring hydrophilicity and surface energy just prior to implantation, clinicians can potentially double the osseointegration potential of implants. For example, BIC has been shown to increase from 58% to over 90%, and biomechanical anchorage more than doubled, when using freshly prepared implants compared to 4-week-old counterparts. This underscores the transformative value of managing surface aging in the clinical setting.

Another key clinical implication is the inequality of implants in their biological performance, depending on their age. Two implants with identical design and surface specifications may yield different healing outcomes if one has been aged significantly longer than the other. For clinicians, this highlights the need for not only careful inventory control but also reconsideration of how implant shelf life is defined and communicated. Standardizing biological performance by addressing surface aging may help ensure more consistent and predictable clinical outcomes, reducing early failures and enhancing long-term success.

### Overlooked variable: biological aging and its impact on implant research

The age of experimental implant materials—referring to the time elapsed since their fabrication—has rarely been documented, let alone standardized, in implant-related studies. This applies to titanium, titanium alloys, chromium-cobalt alloys, and other commonly tested materials. Despite numerous studies comparing the bioactivity of different implant materials, surface topographies, and surface treatments, the unaccounted aging of samples presents a major confounding factor that can skew results and compromise the validity of conclusions.

For example, when comparing machined surfaces with microrough surfaces produced by acid-etching, it is likely that the latter were prepared more recently for experimental purposes, while the machined surfaces were older stock. This potential disparity in material age is especially problematic given the well-documented biological aging of titanium, where surface bioactivity can degrade significantly within weeks of exposure to air.

Studies have shown that biological aging results in a twofold–threefold reduction in cell attachment and alkaline phosphatase (ALP) activity [9, 11, 153], and up to tenfold or more differences in physicochemical properties like surface hydrophilicity [9, 11, 134, 153]. Even a modest difference, such as four weeks between the preparation of two samples, can produce biologically significant discrepancies. One simulative study modeling progressive carbon accumulation reported that a twofold increase in surface carbon contamination resulted in a threefold reduction in osteoblast attachment, spreading area, and ALP activity [158].

Recognizing the role of biological aging in implant materials calls for a paradigm shift in experimental design. Future implant studies should document and standardize the age of all materials used—especially control and test groups—to ensure accurate, reproducible, and interpretable results. This insight into biological aging not only enhances the scientific rigor of implant research but also lays the groundwork for new methodological standards in the field. Furthermore, because the hydrophilic/hydrophobic state and surface carbon content are both tightly linked to the factor of time, future studies should include these parameters as essential descriptors of titanium surface condition.

### Pre-photofunctionalization attempts to prevent biological aging: lessons and limitations

Because the physicochemical changes result from unavoidable environmental interactions, finding an effective method to prevent aging at the manufacturing or implant storage stage has proven extremely challenging. Nevertheless, several approaches have been explored to mitigate the decline in bioactivity that occurs during storage.

One such attempt involved coating freshly prepared titanium surfaces with a nonvolatile liquid buffer, specifically 4-(2-Hydroxyethyl)-1-piperazineethanesulfonic acid (HEPES) [140]. HEPES, a biologically safe zwitterionic buffer, was applied as a thin (~10 µm) film onto freshly made, superhydrophilic titanium surfaces. The coating retained surface hydrophilicity for at least 3 months and preserved osteoblast attachment, ALP activity, and calcium deposition at levels comparable to freshly prepared titanium. In vivo, HEPES-coated titanium exhibited 2.5 times stronger bone integration than 3-month-old uncoated implants, equating to the strength seen in new implants. However, this method comes with a caveat—coating with HEPES, a carbon-containing molecule, fundamentally altered the titanium surface. As such, it did not truly prevent carbon adsorption but replaced it with a different organic layer, raising concerns about biocompatibility.

Another approach attempted to modulate surface topography to slow biological aging. Titanium surfaces combining micro- and nano-scale structures—created through acid-etching followed by TiO₂ nanonodular self-assembly—maintained biological activity longer than microrough-only surfaces [130]. Although these hybrid surfaces lost hydrophilicity even faster than microrough ones, they retained high levels of osteoblastic activity for several days, suggesting that hydrophilicity is not the sole determinant of biological performance. The extended bioactivity was abolished by exposure to monovalent anions such as chloride, implicating the maintenance of positive surface electrostatic charge as a critical factor. However, the mechanism of electrostatic retention and its degradation over time remains poorly understood, limiting its practical use as a preventive strategy.

A widely explored strategy has been storing titanium in saline solution immediately after surface preparation to maintain hydrophilicity. While water spreads across the saline-stored titanium surface, creating the appearance of hydrophilicity, this may be a misleading phenomenon. True hydrophilicity is defined by the dynamic interaction between water molecules and a solid surface—specifically, the interfacial energy between unlike molecules. In contrast, the behavior of saline-coated titanium may reflect water–water cohesion, where the cohesive forces among water molecules dominate due to the pre-wetted state. This distinction is critical: saline storage may merely mask hydrophobicity rather than prevent it. Moreover, saline storage did not prevent surface carbon contamination, with up to 33.4% of the surface still covered in carbon [159]. Biological outcomes also appeared limited—while early-stage osteoblast differentiation markers such as ALP and collagen I were upregulated, late-stage markers like osteocalcin remained unaffected [159]. More importantly, the recruitment and attachment of cells—typically enhanced on genuinely hydrophilic surfaces—were actually diminished on saline-stored titanium. Surface analysis revealed nano-deposits suggestive of crystallized NaCl with increased Sa, indicating that the surface is not simply hydrated titanium but chemically modified. This alteration, coupled with changes in topography and roughness, complicates the interpretation of biological outcomes and raises concerns about attributing observed effects solely to preserved hydrophilicity.

In summary, while several inventive techniques have been proposed to slow or mitigate the biological aging of titanium, all of them involve altering the original surface either chemically or physically. Critically, no method to date has succeeded in preserving both key properties—hydrophilicity and carbon-free surface—on genuine titanium surfaces. These limitations underscore the significance of UV photofunctionalization as the first truly effective, non-coating, non-invasive approach that restores the native, high-energy state of titanium just before clinical use.

### Early attempt to restore the age-degraded capability of implant materials

This section introduces early efforts to reverse the effects of biological aging on titanium surfaces—an attempt to "turn back time" after preventive strategies proved insufficient. Since both the loss of hydrophilicity and the accumulation of surface carbon are associated with reductions in surface energy, initial restoration strategies focused on re-energizing the titanium surface.

One of the first approaches tested was the use of gamma irradiation [160]. Gamma rays are high-energy electromagnetic waves with the shortest wavelengths in the spectrum, known for their deep penetration and use in sterilizing medical and dental devices. In fact, the majority of commercially available dental implants are gamma-sterilized at doses typically ranging from 25 to 35 kGy, with 25 kGy being the industry standard to achieve a sterility assurance level (SAL) of 10^−6^. In the referenced study, aged acid-etched titanium disks were subjected to 30 kGy of gamma irradiation. Post-treatment, the titanium surface exhibited a significant increase in hydrophilicity, with the contact angle reduced to 10°, compared to over 70° in untreated, aged titanium. In vitro testing showed that osteoblast attachment on gamma-treated surfaces was 20–30% greater, and in vivo biomechanical assessments demonstrated a 30–40% increase in bone–implant integration strength.

Despite these improvements, the level of biological restoration achieved by gamma irradiation did not fully match that of freshly prepared titanium surfaces. The gamma-treated surfaces became hydrophilic but not superhydrophilic, and although the atomic carbon content was significantly reduced, it remained higher than that of new titanium. Additional experiments using even higher doses—up to 100 kGy—did not result in further meaningful reductions in surface carbon, highlighting a limitation in the ability of gamma radiation to fully reverse the physicochemical degradation associated with biological aging. Moreover, gamma-treated surfaces remain susceptible to re-aging unless they are immediately used in clinical settings. This introduces a critical and unresolved concern regarding the practicality and durability of this approach. In summary, while gamma irradiation provides partial restoration of biological function and surface energy, its inability to fully regenerate the original, pristine state of titanium underscores the need for more effective and durable rejuvenation strategies.

### UV photofunctionalization: a complete reset of titanium’s biological clock

#### Reversing time: physicochemical restoration by UV photofunctionalization

UV photofunctionalization has redefined the capabilities of titanium by fully reversing its biological aging and restoring its surface physicochemistry [9, 34, 116, 132, 154, 157, 161, 162]. By exposing titanium to UV light (100–400 nm), hydrocarbon contaminants or carbon pellicle are dramatically reduced—atomic carbon content decreases from 40–75 to 15–20%—through direct bond dissociation, oxygen radical formation, and TiO₂-mediated photocatalysis [147, 163]. This decontamination process restores the pristine, high-energy state of the titanium surface.

One critical outcome is the return of surface electrostatic charge. Fresh titanium is electropositive, but aging and hydrocarbon accumulation reverse this charge to negative [148–150, 164]. UV treatment not only recovers the original charge but amplifies it [151, 164, 165], enhancing protein adsorption via spontaneous electrostatic attraction rather than relying on ionic bridges [34, 151, 165]. For example, UV-treated titanium absorbed significantly more albumin at neutral pH than untreated surfaces [165].

**Fig. 6:**
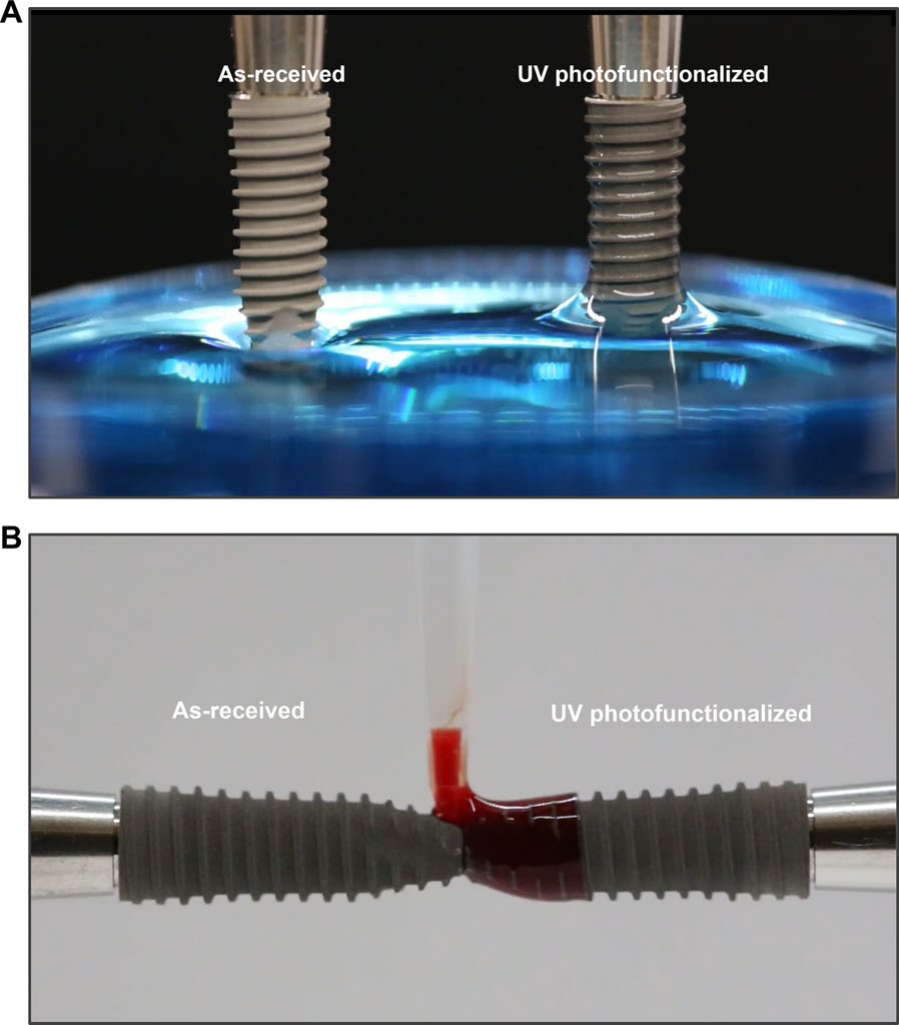
High-energy, superhydrophilic dental implants induced by UV photofunctionalization. Commercial dental implants were treated with vacuum UV (VUV) light for 1 min and tested for wettability using distilled water (**A**) and whole blood (**B**). **A** When implants were brought into contact with water, the as-received implant exhibited a funnel-shaped water profile, indicating strong hydrophobicity and liquid repellence. In contrast, UV-photofunctionalized implants immediately absorbed water, which climbed along the thread surfaces, demonstrating superhydrophilicity. **B** Whole blood placed between an as-received and a UV-treated implant migrated exclusively toward the UV-photofunctionalized implant, leaving the as-received surface untouched. This selective flow reveals the strong liquid-attracting capacity of UV-treated titanium and highlights its high surface energy

Another hallmark of UV photofunctionalization is the transformation from hydrophobicity to superhydrophilicity. UV-treated surfaces exhibit contact angles at or near 0°, even after months or years of aging, and across various surface conditions, including alcohol-cleaned, autoclaved, or saline-soaked specimens [8, 10, 12, 18, 132, 135, 141, 166, 167]. This superhydrophilicity enhances biological interactions by maximizing the contact area between titanium and its surrounding environment, while also promoting the attraction of polar molecules critical for cellular and molecular responses. These high-energy surfaces stand in stark contrast to conventional, as-received implants, as clearly demonstrated in commercial implant comparisons (Fig. 6), where water and blood behavior vividly illustrate the dramatic shift in surface energy and biological affinity.

#### Enhanced osteogenic cell response to UV-photofunctionalized titanium

UV photofunctionalization renders titanium superhydrophilic, enabling strong attraction of blood and proteins and promoting uniform fluid spread for better delivery of bioactive agents [9, 10, 34, 116, 168]. RGD-based studies suggest that UV-treated titanium gains a positive surface charge, allowing direct interaction with negatively charged biomolecules without the need for divalent cation bridges [151, 165]. Therefore, UV treatment significantly accelerates and increases protein adsorption. Albumin and fibronectin binding rose five- to six-fold within two hours of incubation compared to untreated surfaces [34]. Even after 24 h, UV-treated surfaces continued to exhibit higher protein accumulation—approximately double that of controls [82].

These effects translate into enhanced cell behavior: osteoblast attachment increased three- to five-fold within three hours, and the difference remained significant after 24 h. UV treatment also mitigates the lower cell recruitment typically seen on microrough surfaces [38]. UV-treated titanium promotes osteoblast proliferation and function. On acid-etched titanium, osteoblast density and BrdU-based proliferation were 200% higher on UV-treated surfaces after 4 days [34]. Osteogenic markers also improved, with nearly twice the ALP activity at day 7 and mineralization at day 14. These findings were consistent in other osteogenic models, including periosteal- and human mesenchymal stromal cell-derived osteoblasts [116, 169].

The effect of UV photofunctionalization extends to other materials, such as zirconia, where increased cell attachment and proliferation have been observed. These improvements are linked to increased hydrophilicity and reduced surface hydrocarbons [73], underscoring the broader applicability of UV photofunctionalization in implant biomaterials.

#### In vivo excellence of UV-photofunctionalized implants: from partial osseointegration to complete osseointegration

##### Reprogramming bone healing: from distant to contact osteogenesis

Osseointegration traditionally, as shown around machined implants, occurs through distance osteogenesis, where bone slowly grows inward from surrounding tissues [115]. In contrast, contact osteogenesis—where bone formation initiates directly at the implant surface—is the ideal, as it establishes a more immediate and intimate bond between bone and implant [115]. However, current microrough titanium implants fail to consistently trigger this favorable response.

Fluorescent histological analysis in a canine model revealed that untreated titanium implants predominantly induced distant osteogenesis, with alizarin red—a mineralization marker—detected only in the peripheral bone, not at the implant interface [15]. This spatial absence of mineralization indicated delayed and insufficient integration. In contrast, UV-treated implants showed robust alizarin red deposition across the entire implant interface, reflecting strong and early-stage contact osteogenesis.

Further evidence comes from a rat femur model with 0.5 mm intentional peri-implant gaps [169]. Only UV-treated implants initiated bone formation from both the implant surface and surrounding bone, whereas control implants supported bone growth exclusively from the distant bone. This dual-direction healing highlights UV’s ability to reprogram the healing pathway toward effective and complete osseointegration by eliminating hydrocarbon contamination and restoring surface energy and electrostatic cell attraction.

##### Near-perfect BIC: achieving the unattainable

The percentage of bone–implant contact (BIC) determines how securely the implant integrates into the surrounding bone. In theory, a higher BIC enhances the mechanical interlocking and support at the interface, enabling superior load transfer and minimizing micromovement. This translates into more uniform stress distribution, reduced stress concentration zones, and lower risk of bone resorption. Biomechanically, increased BIC is associated with improved interfacial stiffness and altered local elastic modulus, enhancing not just the yield strength but also the system’s ability to absorb energy and resist fatigue. The increased yield strength and deformation tolerance of the peri-implant complex directly contribute to the interfacial energy, an indicator for long-term mechanical stability and preservation of the supporting bone structure under functional loading. However, most commercially available titanium surfaces have a BIC ceiling of approximately 45 ± 16% [30, 31, 136–138], highlighting a significant limitation in current implant technologies.

UV photofunctionalization has been shown to break through this performance plateau. In a rat femur model, UV-treated implants achieved a BIC of 98.2% at week 4 of healing—nearly double the 53% observed in untreated controls [34]. Notably, soft tissue penetration at the bone–implant interface dropped to below 1% in UV-treated implants, compared to 21% in the control group, indicating a pronounced shift toward contact osteogenesis. Bone volume at the interface also increased by 80%, further contributing to the enhanced integration. This near-complete BIC was the result of both quantitatively greater bone formation and qualitatively superior contact osteogenesis. Together, these effects establish UV photofunctionalization as a transformative strategy in achieving previously unattainable levels of implant integration.

These results surpassed the 92% BIC seen with freshly prepared surfaces [9], showing that UV treatment not only restores but surpasses baseline osseointegration capacity. Remarkably, studies in rabbits found that UV-treated implants achieved higher bone volume [170] and that the UV-photofunctionalized machined implants showed higher BIC than untreated microrough implants [171], suggesting that the physicochemical effects often exceed topographical ones. This study also revealed that bone formation in the distant area was increased around UV photofunctionalized implants. In dog models, UV-treated implants demonstrated 50–100% increases in bone volume across both cortical and marrow zones, along with denser mineralization near the implant collar [15].

##### Biomechanical benefits: faster, stronger, and more reliable integration

The biological advantages conferred by UV treatment translate directly into superior mechanical performance. In push-in tests, UV-treated implants exhibited osseointegration strength three times greater than that of untreated controls after just 2 weeks [34]. Remarkably, untreated implants required eight weeks to reach the same strength level attained by UV-treated implants in only two weeks—representing a fourfold acceleration in the healing process.

In a dog model, the removal torque of UV-treated implants was 150% that of untreated implants [15]. Even more impressively, the variation in removal torque values shrank dramatically—from 22% to just 3%—indicating far more consistent and reliable integration with UV-treated surfaces.

#### Load-bearing success under adverse conditions: functional and mechanical endurance of UV-photofunctionalized titanium

A critical measure of osseointegration is how well implants perform under unfavorable conditions, especially in situations that mimic real-life clinical challenges. UV photofunctionalization has demonstrated not only accelerated osseointegration but also mechanical robustness under biologically and biomechanically adverse conditions. One of the most compelling models to evaluate this is the use of short implants, where limited surface area inherently reduces bone contact and compromises stability [172]. In a rat femur model, 1.2 mm short implants with UV treatment outperformed even longer, 2.0 mm untreated implants, achieving a 100% higher push-in test value at 2 weeks and equivalent strength by week 8. These findings suggest that UV treatment can fully compensate for the structural limitations of short implants, a frequent challenge in compromised bone volume cases and provide a novel strategy for minimum invasive surgery.

Systemic conditions known to impair osseointegration—including osteoporosis, diabetes, and advanced age—were also modeled to assess the reliability of UV-treated implants. In ovariectomized (OVX) rats simulating postmenopausal osteoporosis, osteoblasts displayed significantly reduced functional activity, bone density, and integration potential on untreated titanium [22]. However, when UV-treated titanium was used, osteoblasts from OVX rats showed restored and even enhanced attachment, proliferation, and osteogenic activity, achieving levels comparable to or exceeding those from healthy control animals. In vivo, the mechanical strength of bone–implant integration for UV-treated implants was 80% greater than controls in OVX rats, surpassing even the values seen in healthy sham-operated rats. These findings confirm the capacity of UV photofunctionalization to overcome systemic skeletal deficiencies.

In a rat model of type 2 diabetes—characterized by late-onset hyperglycemia and obesity—UV photofunctionalization similarly overcame systemic impairment of osseointegration [20]. Photofunctionalized implants exhibited osseointegration strength that was 1.8 times greater than untreated implants at two weeks and nearly three times greater at four weeks. Remarkably, their integration strength in diabetic animals exceeded that of untreated implants in healthy controls throughout the healing period. Surface analysis revealed extensive coverage of calcium- and phosphorus-rich tissue on UV-treated implants, indicative of robust mineralized bone formation. These results underscore the therapeutic potential of UV photofunctionalization in reversing diabetes-induced compromise in implant integration.

Aging, which is often associated with diminished cellular responsiveness and impaired wound healing, also presented a meaningful biological challenge. Bone marrow-derived osteoblasts from aged rats (15 months) showed substantial declines in functional capabilities compared to younger counterparts (8 weeks) [23]. Yet, when cultured on UV-treated titanium, these cells exhibited a marked increase in attachment and osteoblastic functions. Correspondingly, in vivo testing revealed that UV-treated implants placed in aged rats experienced a 40% higher integration strength than untreated implants. Elemental mapping further confirmed more robust bone formation around UV-treated surfaces, characterized by stronger calcium and phosphate signals, hallmarks of mature bone matrix deposition.

UV photofunctionalization also showed remarkable performance under mechanical stress conditions, such as immediate loading [167]. In this rat model, implants were subjected to a constant lateral force of 0.46 N immediately after placement. The osseointegration success rate of untreated implants was only 28.6%, whereas UV-treated implants achieved a 100% success rate. Not only was integration preserved, but the strength of osseointegration was 2.4 times higher in the UV group, and implant tilting—an indicator of mechanical failure—was reduced by half. These results underscore the ability of UV-treated surfaces to stabilize and sustain integration during immediate and early functional loading, a crucial factor in clinical protocols seeking faster recovery and reduced treatment times.

In summary, UV photofunctionalization has demonstrated reliable mechanical anchorage and biological integration across a diverse range of challenging conditions. Whether facing systemic impairments such as osteoporosis, diabetes, and aging, or mechanical threats like immediate loading and limited implant geometry, UV treatment consistently enhances both the success rate and mechanical strength of osseointegration. This robustness underscores its translational potential in clinical cases where traditional implant approaches often fall short.

#### Generalizability of UV photofunctionalization across titanium surface topographies

The effects of UV photofunctionalization have been demonstrated across a wide range of titanium surface topographies beyond acid-etched microrough surfaces. These include machined smooth surfaces, sand-blasted surfaces, hydrofluoric acid-etched surfaces, and nano-featured surfaces [34, 89, 126, 139]. Regardless of the initial topography, UV treatment consistently rendered these surfaces superhydrophilic and significantly reduced surface carbon content. In both in vitro and in vivo studies, UV activation led to notable improvements in cellular and bone responses. Osteoblast attachment, for instance, increased by two- to threefold across different surface types following UV treatment.

However, while the beneficial effects of UV activation are broadly applicable, their magnitude appears to be surface-specific. That is, the degree of enhancement in osseointegration is influenced by the baseline performance of the original surface. For example, after two weeks of healing, UV-treated machined implants showed a twofold increase in bone integration strength—but the absolute value reached only 11 N, which remained lower than the 12 N seen in untreated acid-etched implants [34]. This highlights a common misconception: UV photofunctionalization does not elevate every surface to a uniformly high level of performance. Rather, its effectiveness scales with the inherent bioactivity of the underlying topography. A general trend has emerged—rougher surfaces tend to exhibit greater gains from UV treatment.

#### Summary: redefining the bone–implant interface

A systematic review of 34 animal studies concluded that photofunctionalization significantly enhances osseointegration [173]. Similarly, a meta-analysis of seven clinical studies found that UV-treated implants demonstrated an accelerated rate of osseointegration establishment [174]. A comprehensive narrative review further reported that UV photofunctionalization improves in vitro and in vivo outcomes by enhancing cellular migration, attachment, and proliferation—ultimately promoting both osseointegration and coronal soft tissue sealing [175].

**Fig. 7:**
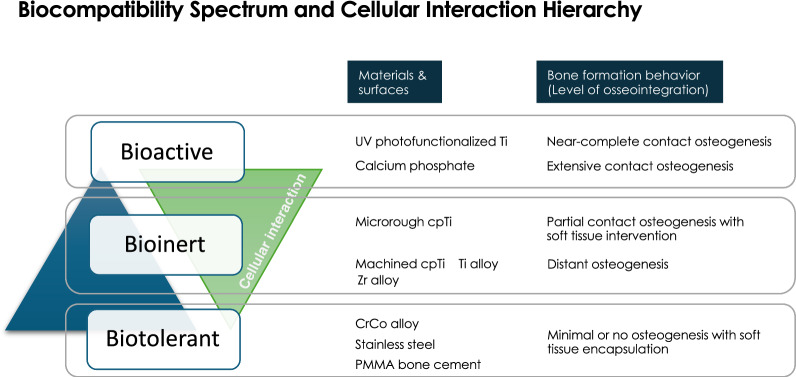
Biocompatibility spectrum and cellular interaction hierarchy for biomaterials. This conceptual diagram classifies biomaterials along two interrelated hierarchies: biocompatibility (*blue upright pyramid*) and cellular interaction (*green inverted pyramid*). The biocompatibility spectrum ranges from biotolerant materials (e.g., stainless steel, cobalt-chrome alloys, PMMA bone cement), to bioinert (e.g., conventional titanium and zirconia), and ultimately to bioactive materials (e.g., hydroxyapatite and UV-photofunctionalized titanium). Lower tiers of the pyramid are associated with greater cytotoxicity and minimal tissue integration, while higher tiers reflect increasing biocompatibility and affinity for biological tissues. Titanium has long been classified as bioinert due to the passive, stable nature of its surface oxide layer. However, UV photofunctionalization repositions titanium into the bioactive category, driven by the removal of hydrocarbon contamination, restoration of superhydrophilicity, and dramatic increase in surface energy (refer to Fig. 6). These physicochemical enhancements lead to robust cellular attachment, superior osteoconductivity, and impeccable contact osteogenesis—a phenotype culminating in what is termed superosseointegration, marked by near-complete bone–implant contact and integration. The green inverted pyramid illustrates the cellular interaction hierarchy, where materials positioned higher show greater biological activity and direct engagement with surrounding cells. UV-treated titanium is placed at the apex of this hierarchy, representing its unmatched capacity for high-fidelity cellular interaction and functional tissue integration. The diagram also incorporates a gradation of bone formation behavior (levels of osseointegration), defined as: (i) Near-complete contact osteogenesis—seamless bone-to-implant integration with minimal interfacial gaps. (ii) Extensive contact osteogenesis—strong bone formation directly along the implant surface. (iii) Partial contact osteogenesis with soft tissue intervention—mixed bone and soft tissue response at the interface. (iv) Distant osteogenesis—bone formation occurs away from the implant, with limited surface integration. (v) Minimal or no osteogenesis with soft tissue encapsulation—poor integration; fibrous tissue dominates the interface. Together, these hierarchies and behavioral descriptors underscore the biological advancement conferred by UV photofunctionalization and its potential to redefine the clinical expectations of titanium-based implants

The in vivo findings presented in the current review offer compelling support: UV photofunctionalization fundamentally redefines the behavior of titanium implants. It transforms titanium from a passive, bioinert material into a bioactive surface capable of actively recruiting osteogenic cells, promoting contact osteogenesis, and achieving near-complete bone–implant integration. This high-energy transformation is driven by the elimination of the hydrocarbon pellicle and the restoration of surface hydrophilicity (Fig. 6). This synergistic enhancement of surface energy and cellular response establishes what can be termed superosseointegration [13, 135]—a new standard in implant performance. These findings underscore the critical role of time-governed physicochemical changes in titanium surfaces and unlock a new understanding of titanium's potential. As such, UV-photofunctionalized titanium should be reclassified from bioinert to bioactive, as conceptually illustrated in Fig. 7.

## Discussion

**Fig. 8:**
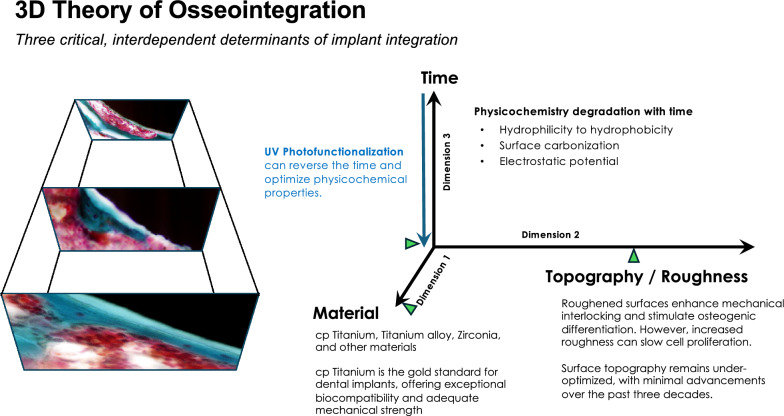
The 3D Theory of Osseointegration: A multidimensional model for implant integration. This conceptual 3D diagram illustrates the three interdependent and indispensable dimensions that govern implant osseointegration, each mapped to a spatial axis—material, topography, and time—to highlight their combined influence on integration outcomes. Together, these dimensions define the Implant Domain—a dynamic space in which osseointegration is not merely a material-dependent phenomenon but the result of synergistic, time-sensitive surface optimization. The 3D Theory underscores that true implant success requires not only optimal material selection and surface topography but also proactive control of time-dependent surface degradation, placing surface aging and UV photofunctionalization as crucial, controllable factors in maximizing implant performance. Green arrowheads in the diagram indicate the current levels of optimization based on findings from the present review. Notably, material composition (Dimension 1) and physicochemical property management (Dimension 3) have been advanced to their highest levels through the use of titanium and UV-mediated surface reactivation, respectively. In contrast, surface topography (Dimension 2) remains underdeveloped. Despite the widespread adoption of microrough surfaces, no significant progress has been made in over three decades. Furthermore, no clear quantitative correlation has been established between topographic metrics and osseointegration outcomes. This gap highlights the urgent need for a quantitative, engineering-driven approach to surface design—one that transcends empirical roughening and moves toward precision-based, biologically informed topographic optimization

This review explored the three core surface characteristics of dental implants—material composition, surface topography, and physicochemical properties—as interdependent dimensions that govern osseointegration. While often treated as separate domains, these factors interact in complex, sometimes synergistic ways. Optimizing each individually—and in relation to one another—is essential for advancing implant design and clinical outcomes. Crucially, physicochemical properties are governed by time, introducing temporal dynamics as a “third dimension” in implant surface science (Fig. 8).

Material composition has largely reached a consensus: cpTi remains the gold standard, offering unmatched biocompatibility and mechanical properties. In contrast, physicochemical properties have only recently been recognized as dynamic. Titanium surfaces experience biological aging, defined by time-dependent accumulation of hydrocarbon contaminants and a corresponding decline in surface energy—beginning immediately after manufacturing. This degradation compromises biological performance. However, this phenomenon has spurred the development of UV photofunctionalization, based on the revelation that titanium is not permanently inert. Instead, its biological activity can be restored by reversing the effects of aging. Through UV treatment, aged titanium can be rejuvenated to a bioactive state, reshaping our understanding of how implant surfaces interact with tissue (Fig. 7).

Among the most compelling indicators of surface optimization are superhydrophilicity—a contact angle approaching 0°—and surface carbon levels reduced to approximately 15%. These parameters mark the functional ideal of titanium surface conditioning. While conventional UV systems required prolonged exposure to achieve these results, the advent of vacuum UV (VUV) systems has dramatically shortened treatment time. VUV devices have shown that complete decarbonization and superhydrophilicity can be achieved within just 1 min [13, 132, 145–147, 161, 176]. This technological leap has redefined the feasibility and efficiency of surface activation protocols in clinical settings.

In contrast, surface topography remains the least optimized of the three dimensions. While today’s microrough surfaces are a major advance over the smooth machined surfaces of earlier generations, their development was largely empirical, not guided by systematic engineering principles [43]. There is no established framework identifying the "optimal" topography for osseointegration. We lack reliable correlations between surface parameters—such as roughness, pattern orientation, or scale—and biological responses. The question remains: How far can roughness be increased before it ceases to provide biological benefit? Furthermore, as shown in Table 1, topographies at different scales—nano, micro, and meso—play distinct biological roles. However, current implant surfaces are not biologically target-driven designs, but rather products of conventional manufacturing techniques. We must move beyond treating topography as a passive feature and begin actively engineering it to meet specific biological goals, such as overcoming the dichotomous behavior of osteoblast proliferation versus differentiation (Fig. 2). Even the basic correlation between surface roughness and mechanical interlocking remains underexplored in implant science. A mechanistic, design-oriented approach that integrates biomechanics, materials science, and cellular biology is urgently needed. This approach would allow us to define topographies that predictably enhance cell attachment, promote osteogenesis, or inhibit bacterial adhesion.

A key insight from this review is that the three surface characteristics—material, topography, and physicochemistry—are not always synergistic, nor are they entirely independent. For instance, a favorable topography cannot fully compensate for poor surface energy, and vice versa. Under standard conditions, rougher surfaces tend to be more hydrophobic, yet become more hydrophilic after UV treatment. This shows that physicochemical properties are modifiable—capable of enhancement or deterioration—and that their behavior depends on surface morphology and material constraints. Material composition, in turn, sets the limits for what is chemically and topographically feasible. Thus, while synergy among these three dimensions is the ultimate goal, it cannot be assumed. It must be engineered and verified. Each factor should be independently optimized, and their interdependencies systematically mapped through experimental data and computational modeling.

This review primarily focused on biological responses to implants, particularly those involving cellular behavior, osseointegration dynamics, and surface domains. Clinical studies, including patient and surgical factors, and long-term outcomes, were not reviewed and thus fall outside the scope of this analysis. Likewise, purely material science or engineering-based studies—those not directly addressing biological performance—were excluded, although they may contain emerging technologies with potential clinical relevance. As a result, novel surface treatment techniques or materials under early development may not have been captured in this framework. Furthermore, while the 3D Theory of Osseointegration offers a conceptual foundation and strategic direction for implant surface design, its practical implementation must be evaluated through the lens of technological feasibility, commercial scalability, long-term stability, and cost-effectiveness. Bridging the gap between theoretical optimization and clinical application remains a critical challenge, necessitating interdisciplinary collaboration and translational research efforts.

This review emphasizes that the future of implant surface design lies not in the coexistence, but in the integration of the three surface dimensions. Topography remains the next frontier, as it defines the physical landscape where cells interact with the implant. If material composition provides the foundational platform, and physicochemical properties dictate biological affinity, then topography forms the tangible interface with the biological environment. Organized under the framework of the 3D theory of osseointegration (Fig. 8), we now possess the conceptual and technological tools to systematically design, evaluate, and implement implant surfaces with clear biological purposes. The next generation of implants will not only be more biocompatible but will be intelligent by design, optimized to engage with biological systems in a deliberate, dynamic, and time-sensitive manner.

## Conclusion

This review presents a unifying 3D Theory of Osseointegration, in which material composition (dimension 1), surface topography (dimension 2), and time-dependent physicochemical properties (dimension 3) are understood as interdependent and dynamic determinants of implant integration. While titanium and its alloys have long set the benchmark for material biocompatibility, the discovery of biological aging and the development of UV photofunctionalization have redefined how surface optimization must be approached. Time—once an overlooked variable—now emerges as a critical determinant of biological readiness, governing surface energy, hydrophilicity, and carbon contamination. Even the most ideal material and topography lose biological effectiveness if the time-induced degradation of surface physicochemistry is ignored.

UV-based physicochemical rejuvenation has proven that surface bioactivity is not fixed but recoverable, challenging the long-standing notion of titanium’s inertness. This insight establishes a new paradigm in which surface function can be actively restored or enhanced, even after manufacturing and storage. Titanium is no longer seen as a bioinert material, but one whose surface bioactivity can be activated and optimized. Moreover, these principles have shown relevance across materials beyond titanium, reinforcing their universal applicability.

However, surface topography remains the least engineered dimension. Despite its pivotal biological role and the widespread adoption of microrough surfaces, its development has largely lacked mechanistic or quantitative guidance. The field must now move beyond empirical surface designs toward intentional, model-driven topographical engineering, grounded in biological validation and precision.

Most importantly, these three dimensions must not be treated in isolation. Material provides the foundational platform, topography shapes the structural interface, and physicochemical properties govern the biological dialogue. Their synergy must be intentionally designed—not merely assumed. The 3D Theory of Osseointegration thus serves as a blueprint for the next generation of implant surfaces—surfaces that are not only biocompatible, but biologically purposeful, temporally responsive, and precisely engineered. Within this framework, osseointegration is redefined—not as a passive biological response, but as an actively controllable process, guided by deliberate, intelligent design.

## Data Availability

No datasets were generated or analysed during the current study.

## Abbreviations

BIC: Bone–implant contact
cpTi: Commercially pure titanium
CoCr: Cobalt–chromium
Y-TZP: Yttria-stabilized tetragonal zirconia polycrystal
HIP: Hot isostatic pressing
TPS: Titanium plasma-sprayed
ALP: Alkaline phosphatase
CFD: Computational fluid dynamics
HEPES: 4-(2-Hydroxyethyl)-1-piperazineethanesulfonic acid

## References

[1] Albrektsson T, Branemark PI, Hansson HA, Lindstrom J. Osseointegrated titanium implants. Requirements for ensuring a long-lasting, direct bone-to-implant anchorage in man. Acta Orthop Scand. 1981;52(2):155–70. 7246093 10.3109/17453678108991776

[2] Cooper LF. A role for surface topography in creating and maintaining bone at titanium endosseous implants. J Prosthet Dent. 2000;84(5):522–34. 11105008 10.1067/mpr.2000.111966

[3] Cooper LF, Masuda T, Yliheikkila PK, Felton DA. Generalizations regarding the process and phenomenon of osseointegration. Part II. In vitro studies. Int J Oral Maxillofac Implants. 1998;13(2):163–74. 9581401

[4] Wennerberg A, Albrektsson T. On implant surfaces: a review of current knowledge and opinions. Int J Oral Maxillofac Implants. 2010;25(1):63–74. 20209188

[5] Jokstad A, Sanz M, Ogawa T, Bassi F, Levin L, Wennerberg A, et al. A systematic review of the role of implant design in the rehabilitation of the Edentulous Maxilla. Int J Oral Maxillofac Implants. 2016;31(Suppl):s43-99. 27228254 10.11607/jomi.16suppl.g2

[6] Att W, Tsukimura N, Suzuki T, Ogawa T. Effect of supramicron roughness characteristics produced by 1- and 2-step acid etching on the osseointegration capability of titanium. Int J Oral Maxillofac Implants. 2007;22(5):719–28. 17974105

[7] Kubo K, Att W, Yamada M, Ohmi K, Tsukimura N, Suzuki T, et al. Microtopography of titanium suppresses osteoblastic differentiation but enhances chondroblastic differentiation of rat femoral periosteum-derived cells. J Biomed Mater Res A. 2008;87(2):380–91. 18181115 10.1002/jbm.a.31791

[8] Att W, Hori N, Iwasa F, Yamada M, Ueno T, Ogawa T. The effect of UV-photofunctionalization on the time-related bioactivity of titanium and chromium-cobalt alloys. Biomaterials. 2009;30(26):4268–76. 19473697 10.1016/j.biomaterials.2009.04.048

[9] Att W, Hori N, Takeuchi M, Ouyang J, Yang Y, Anpo M, et al. Time-dependent degradation of titanium osteoconductivity: an implication of biological aging of implant materials. Biomaterials. 2009;30(29):5352–63. 19595450 10.1016/j.biomaterials.2009.06.040

[10] Att W, Ogawa T. Biological aging of implant surfaces and their restoration with ultraviolet light treatment: a novel understanding of osseointegration. Int J Oral Maxillofac Implants. 2012;27(4):753–61. 22848875

[11] Lee JH, Ogawa T. The biological aging of titanium implants. Implant Dent. 2012;21(5):415–21. 22968574 10.1097/ID.0b013e31826a51f4

[12] Funato A, Yamada M, Ogawa T. Success rate, healing time, and implant stability of photofunctionalized dental implants. Int J Oral Maxillofac Implants. 2013;28(5):1261–71. 24066316 10.11607/jomi.3263

[13] Park G, Matsuura T, Komatsu K, Ogawa T. Optimizing implant osseointegration, soft tissue responses, and bacterial inhibition: a comprehensive narrative review on the multifaceted approach of the UV photofunctionalization of titanium. J Prosthodont Res. 2024;69(2):136–52. 10.2186/jpr.JPR_D_24_00086. 38853001 10.2186/jpr.JPR_D_24_00086

[14] Park KH, Koak JY, Kim SK, Heo SJ. Wettability and cellular response of UV light irradiated anodized titanium surface. J Adv Prosthodont. 2011;3(2):63–8. 21814613 10.4047/jap.2011.3.2.63 PMC3141120

[15] Pyo SW, Park YB, Moon HS, Lee JH, Ogawa T. Photofunctionalization enhances bone-implant contact, dynamics of interfacial osteogenesis, marginal bone seal, and removal torque value of implants: a dog jawbone study. Implant Dent. 2013;22(6):666–75. 24185466 10.1097/ID.0000000000000003

[16] Saita M, Ikeda T, Yamada M, Kimoto K, Lee MC, Ogawa T. UV photofunctionalization promotes nano-biomimetic apatite deposition on titanium. Int J Nanomed. 2016;11:223–34. 10.2147/IJN.S95249 PMC4716735 26834469

[17] Yamada M, Miyauchi T, Yamamoto A, Iwasa F, Takeuchi M, Anpo M, et al. Enhancement of adhesion strength and cellular stiffness of osteoblasts on mirror-polished titanium surface by UV-photofunctionalization. Acta Biomater. 2010;6(12):4578–88. 20633705 10.1016/j.actbio.2010.07.010

[18] Kitajima H, Ogawa T. The use of photofunctionalized implants for low or extremely low primary stability cases. Int J Oral Maxillofac Implants. 2016;31(2):439–47. 27004291 10.11607/jomi.4054

[19] Moy PK, Medina D, Shetty V, Aghaloo TL. Dental implant failure rates and associated risk factors. Int J Oral Maxillofac Implants. 2005;20(4):569–77. 16161741

[20] Sugita Y, Honda Y, Kato I, Kubo K, Maeda H, Ogawa T. Role of photofunctionalization in mitigating impaired osseointegration associated with type 2 diabetes in rats. Int J Oral Maxillofac Implants. 2014;29(6):1293–300. 25397793 10.11607/jomi.3480

[21] Hasegawa H, Ozawa S, Hashimoto K, Takeichi T, Ogawa T. Type 2 diabetes impairs implant osseointegration capacity in rats. Int J Oral Maxillofac Implants. 2008;23(2):237–46. 18548919

[22] Taniyama T, Saruta J, Mohammadzadeh Rezaei N, Nakhaei K, Ghassemi A, Hirota M, et al. UV-photofunctionalization of titanium promotes mechanical anchorage in a rat osteoporosis model. Int J Mol Sci. 2020;21(4):1235. 32059603 10.3390/ijms21041235 PMC7072956

[23] Ishijima M, Ghassemi A, Soltanzadeh P, Tanaka M, Nakhaei K, Park W, et al. Effect of UV photofunctionalization on osseointegration in aged rats. Implant Dent. 2016;25(6):744–50. 27513161 10.1097/ID.0000000000000459

[24] Howe MS, Keys W, Richards D. Long-term (10-year) dental implant survival: a systematic review and sensitivity meta-analysis. J Dent. 2019;84:9–21. 30904559 10.1016/j.jdent.2019.03.008

[25] Goyal S, Masood M, Le C, Rajendran Y, Nanjapa S, Vaderhobli R. Comparative bone graft evaluation for dental implant success: an evidence-based review. J Long Term Eff Med Implants. 2021;31(3):33–44. 34369720 10.1615/JLongTermEffMedImplants.2021038292

[26] Giro G, Chambrone L, Goldstein A, Rodrigues JA, Zenobio E, Feres M, et al. Impact of osteoporosis in dental implants: a systematic review. World J Orthop. 2015;6(2):311–5. 25793172 10.5312/wjo.v6.i2.311 PMC4363814

[27] Coelho PG, Marin C, Granato R, Suzuki M. Histomorphologic analysis of 30 plateau root form implants retrieved after 8 to 13 years in function. A human retrieval study. J Biomed Mater Res B Appl Biomater. 2009;91(2):975–9. 19582841 10.1002/jbm.b.31455

[28] Trisi P, Lazzara R, Rebaudi A, Rao W, Testori T, Porter SS. Bone-implant contact on machined and dual acid-etched surfaces after 2 months of healing in the human maxilla. J Periodontol. 2003;74(7):945–56. 12931756 10.1902/jop.2003.74.7.945

[29] Scarano A, Degidi M, Iezzi G, Petrone G, Piattelli A. Correlation between implant stability quotient and bone-implant contact: a retrospective histological and histomorphometrical study of seven titanium implants retrieved from humans. Clin Implant Dent Relat Res. 2006;8(4):218–22. 17100747 10.1111/j.1708-8208.2006.00022.x

[30] Chambrone L, Rincon-Castro MV, Poveda-Marin AE, Diazgranados-Lozano MP, Fajardo-Escolar CE, Bocanegra-Puerta MC, et al. Histological healing outcomes at the bone-titanium interface of loaded and unloaded dental implants placed in humans: a systematic review of controlled clinical trials. Int J Oral Implantol (Berl). 2020;13(4):321–42. 33491365

[31] Ogawa T, Nishimura I. Different bone integration profiles of turned and acid-etched implants associated with modulated expression of extracellular matrix genes. Int J Oral Maxillofac Implants. 2003;18(2):200–10. 12705297

[32] Jokstad A, Braegger U, Brunski JB, Carr AB, Naert I, Wennerberg A. Quality of dental implants. Int Dent J. 2003;53(6 Suppl 2):409–43. 14725421 10.1111/j.1875-595x.2003.tb00918.x

[33] Lopez-Valverde N, Flores-Fraile J, Ramirez JM, Sousa BM, Herrero-Hernandez S, Lopez-Valverde A. Bioactive surfaces vs. conventional surfaces in titanium dental implants: a comparative systematic review. J Clin Med. 2020;9(7):2047. 32610687 10.3390/jcm9072047 PMC7408888

[34] Aita H, Hori N, Takeuchi M, Suzuki T, Yamada M, Anpo M, et al. The effect of ultraviolet functionalization of titanium on integration with bone. Biomaterials. 2009;30(6):1015–25. 19042016 10.1016/j.biomaterials.2008.11.004

[35] Komatsu K, Matsuura T, Cheng J, Kido D, Park W, Ogawa T. Nanofeatured surfaces in dental implants: contemporary insights and impending challenges. Int J Implant Dent. 2024;10(1):34. 38963524 10.1186/s40729-024-00550-1 PMC11224214

[36] Matsuura T, Komatsu K, Cheng J, Park G, Ogawa T. Beyond microroughness: novel approaches to navigate osteoblast activity on implant surfaces. Int J Implant Dent. 2024;10(1):35. 38967690 10.1186/s40729-024-00554-x PMC11226592

[37] Komatsu K, Matsuura T, Suzumura T, Ogawa T. Genome-wide transcriptional responses of osteoblasts to different titanium surface topographies. Mater Today Bio. 2023;23:100852. 38024842 10.1016/j.mtbio.2023.100852 PMC10663851

[38] Hori N, Iwasa F, Ueno T, Takeuchi K, Tsukimura N, Yamada M, et al. Selective cell affinity of biomimetic micro-nano-hybrid structured TiO2 overcomes the biological dilemma of osteoblasts. Dent Mater. 2010;26(4):275–87. 20006380 10.1016/j.dental.2009.11.077

[39] Takeuchi K, Saruwatari L, Nakamura HK, Yang JM, Ogawa T. Enhanced intrinsic biomechanical properties of osteoblastic mineralized tissue on roughened titanium surface. J Biomed Mater Res A. 2005;72A(3):296–305. 10.1002/jbm.a.30227 15654712

[40] Hasegawa M, Saruta J, Hirota M, Taniyama T, Sugita Y, Kubo K, et al. A newly created meso-, micro-, and nano-scale rough titanium surface promotes bone-implant integration. Int J Mol Sci. 2020;21(3):783. 31991761 10.3390/ijms21030783 PMC7036846

[41] Saruta J, Ozawa R, Okubo T, Taleghani SR, Ishijima M, Kitajima H, et al. Biomimetic zirconia with cactus-inspired meso-scale spikes and nano-trabeculae for enhanced bone integration. Int J Mol Sci. 2021. 10.3390/ijms22157969. 34360734 10.3390/ijms22157969 PMC8347469

[42] Rezaei NM, Hasegawa M, Ishijima M, Nakhaei K, Okubo T, Taniyama T, et al. Biological and osseointegration capabilities of hierarchically (meso-/micro-/nano-scale) roughened zirconia. Int J Nanomed. 2018;13:3381–95. 10.2147/IJN.S159955 PMC5997135 29922058

[43] Le Guehennec L, Soueidan A, Layrolle P, Amouriq Y. Surface treatments of titanium dental implants for rapid osseointegration. Dent Mater. 2007;23(7):844–54. 16904738 10.1016/j.dental.2006.06.025

[44] Williams DF. On the mechanisms of biocompatibility. Biomaterials. 2008;29(20):2941–53. 18440630 10.1016/j.biomaterials.2008.04.023

[45] Saulacic N, Bosshardt DD, Bornstein MM, Berner S, Buser D. Bone apposition to a titanium-zirconium alloy implant, as compared to two other titanium-containing implants. Eur Cell Mater. 2012;23:273–86. 22492019 10.22203/ecm.v023a21

[46] Stenport VF, Johansson CB. Evaluations of bone tissue integration to pure and alloyed titanium implants. Clin Implant Dent Relat Res. 2008;10(3):191–9. 18241219 10.1111/j.1708-8208.2007.00077.x

[47] Han CH, Johansson CB, Wennerberg A, Albrektsson T. Quantitative and qualitative investigations of surface enlarged titanium and titanium alloy implants. Clin Oral Implants Res. 1998;9(1):1–10. 9590939 10.1034/j.1600-0501.1998.090101.x

[48] Johansson CB, Han CH, Wennerberg A, Albrektsson T. A quantitative comparison of machined commercially pure titanium and titanium-aluminum-vanadium implants in rabbit bone. Int J Oral Maxillofac Implants. 1998;13(3):315–21. 9638001

[49] Shah FA, Trobos M, Thomsen P, Palmquist A. Commercially pure titanium (cp-Ti) versus titanium alloy (Ti6Al4V) materials as bone anchored implants - Is one truly better than the other? Mater Sci Eng C Mater Biol Appl. 2016;62:960–6. 26952502 10.1016/j.msec.2016.01.032

[50] Lincks J, Boyan BD, Blanchard CR, Lohmann CH, Liu Y, Cochran DL, et al. Response of MG63 osteoblast-like cells to titanium and titanium alloy is dependent on surface roughness and composition. Biomaterials. 1998;19(23):2219–32. 9884063 10.1016/s0142-9612(98)00144-6

[51] Johansson C, Albrektsson T, Thomas T, Sennerby L, Lodding A. Tissue reactions to titanium-6aluminum-4vanadium alloy. Eur J Exp Musculoskel Res. 1992;1:161–9.

[52] Thompson GJ, Puleo DA. Effects of sublethal metal ion concentrations on osteogenic cells derived from bone marrow stromal cells. J Appl Biomater. 1995;6(4):249–58. 8589510 10.1002/jab.770060406

[53] Haynes DR, Rogers SD, Hay S, Pearcy MJ, Howie DW. The differences in toxicity and release of bone-resorbing mediators induced by titanium and cobalt-chromium-alloy wear particles. J Bone Joint Surg Am. 1993;75(6):825–34. 8314823 10.2106/00004623-199306000-00004

[54] Haynes DR, Boyle SJ, Rogers SD, Howie DW, Vernon-Roberts B. Variation in cytokines induced by particles from different prosthetic materials. Clin Orthop Relat Res. 1998;352:223–30. 9678051

[55] Manlapaz M, Maloney WJ, Smith RL. In vitro activation of human fibroblasts by retrieved titanium alloy wear debris. J Orthop Res. 1996;14(3):465–72. 8676260 10.1002/jor.1100140317

[56] Willis J, Li SW, Crean S, Barrak FN. Is titanium alloy Ti-6Al-4 V cytotoxic to gingival fibroblasts-a systematic review. Clin Exp Dent Res. 2021;7(6):1037–44. 34018703 10.1002/cre2.444 PMC8638288

[57] Messous R, Henriques B, Bousbaa H, Silva FS, Teughels W, Souza JCM. Cytotoxic effects of submicron- and nano-scale titanium debris released from dental implants: an integrative review. Clin Oral Invest. 2021;25(4):1627–40. 10.1007/s00784-021-03785-z 33616805

[58] Butz F, Aita H, Wang CJ, Ogawa T. Harder and stiffer bone osseointegrated to roughened titanium. J Dent Res. 2006;85(6):560–5. 16723656 10.1177/154405910608500616

[59] Nakamura H, Saruwatari L, Aita H, Takeuchi K, Ogawa T. Molecular and biomechanical characterization of mineralized tissue by dental pulp cells on titanium. J Dent Res. 2005;84(6):515–20. 15914587 10.1177/154405910508400606

[60] Butz F, Aita H, Takeuchi K, Ogawa T. Enhanced mineralized tissue adhesion to titanium over polystyrene assessed by the nano-scratch test. J Biomed Mater Res A. 2005;74(2):164–70. 15962269 10.1002/jbm.a.30281

[61] Nakamura HK, Butz F, Saruwatari L, Ogawa T. A role for proteoglycans in mineralized tissue-titanium adhesion. J Dent Res. 2007;86(2):147–52. 17251514 10.1177/154405910708600208

[62] Saruwatari L, Aita H, Butz F, Nakamura HK, Ouyang J, Yang Y, et al. Osteoblasts generate harder, stiffer, and more delamination-resistant mineralized tissue on titanium than on polystyrene, associated with distinct tissue micro- and ultrastructure. J Bone Miner Res. 2005;20(11):2002–16. 16234974 10.1359/JBMR.050703

[63] Johansson CB, Sennerby L, Albrektsson T. A removal torque and histomorphometric study of bone tissue reactions to commercially pure titanium and Vitallium implants. Int J Oral Maxillofac Implants. 1991;6(4):437–41. 1820312

[64] Plecko M, Sievert C, Andermatt D, Frigg R, Kronen P, Klein K, et al. Osseointegration and biocompatibility of different metal implants - a comparative experimental investigation in sheep. Bmc Musculoskel Dis. 2012;13:32. 10.1186/1471-2474-13-32 PMC3315746 22400715

[65] Jinno T, Goldberg VM, Davy D, Stevenson S. Osseointegration of surface-blasted implants made of titanium alloy and cobalt-chromium alloy in a rabbit intramedullary model. J Biomed Mater Res. 1998;42(1):20–9. 9740003 10.1002/(sici)1097-4636(199810)42:1<20::aid-jbm4>3.0.co;2-q

[66] Hennig FF, Raithel HJ, Schaller KH, Dohler JR. Nickel-Concentrations, chrom-concentrations and cobalt-concentrations in human tissue and body-fluids of hip-prosthesis patients. J Trace Elem Elect H. 1992;6(4):239–43. 1304233

[67] van Hove RP, Nolte PA, Semeins CM, Klein-Nulend J. Differences in proliferation, differentiation, and cytokine production by bone cells seeded on titanium-nitride and cobalt-chromium-molybdenum surfaces. J Biomater Appl. 2013;28(2):278–87. 22614252 10.1177/0885328212440600

[68] La Budde JK, Orosz JF, Bonfiglio TA, Pellegrini VD Jr. Particulate titanium and cobalt-chrome metallic debris in failed total knee arthroplasty. A quantitative histologic analysis. J Arthroplasty. 1994;9(3):291–304. 8077978 10.1016/0883-5403(94)90084-1

[69] Kitajima H, Komatsu K, Matsuura T, Ozawa R, Saruta J, Taleghani SR, et al. Impact of nano-scale trabecula size on osteoblastic behavior and function in a meso-nano hybrid rough biomimetic zirconia model. J Prosthodont Res. 2023;67(2):288–99. 35858802 10.2186/jpr.JPR_D_22_00015

[70] Kohal RJ, Bachle M, Att W, Chaar S, Altmann B, Renz A, et al. Osteoblast and bone tissue response to surface modified zirconia and titanium implant materials. Dent Mater. 2013;29(7):763–76. 23669198 10.1016/j.dental.2013.04.003

[71] Kohal RJ, Schikofski T, Adolfsson E, Vach K, Patzelt SBM, Nold J, et al. Fracture resistance of a two-piece zirconia implant system after artificial loading and/or hydrothermal aging-an in vitro investigation. J Funct Biomater. 2023;14(12):567. 38132821 10.3390/jfb14120567 PMC10743638

[72] Roehling S, Astasov-Frauenhoffer M, Hauser-Gerspach I, Braissant O, Woelfler H, Waltimo T, et al. In vitro biofilm formation on titanium and zirconia implant surfaces. J Periodontol. 2017;88(3):298–307. 27712464 10.1902/jop.2016.160245

[73] Att W, Takeuchi M, Suzuki T, Kubo K, Anpo M, Ogawa T. Enhanced osteoblast function on ultraviolet light-treated zirconia. Biomaterials. 2009;30(7):1273–80. 19095298 10.1016/j.biomaterials.2008.11.024

[74] Kohal RJ, Wolkewitz M, Hinze M, Han JS, Bachle M, Butz F. Biomechanical and histological behavior of zirconia implants: an experiment in the rat. Clin Oral Implants Res. 2009;20(4):333–9. 19298287 10.1111/j.1600-0501.2008.01656.x

[75] Gahlert M, Rohling S, Wieland M, Eichhorn S, Kuchenhoff H, Kniha H. A comparison study of the osseointegration of zirconia and titanium dental implants. A biomechanical evaluation in the maxilla of pigs. Clin Implant Dent Relat Res. 2010;12(4):297–305. 19438936 10.1111/j.1708-8208.2009.00168.x

[76] Gahlert M, Rohling S, Wieland M, Sprecher CM, Kniha H, Milz S. Osseointegration of zirconia and titanium dental implants: a histological and histomorphometrical study in the maxilla of pigs. Clin Oral Implants Res. 2009;20(11):1247–53. 19531104 10.1111/j.1600-0501.2009.01734.x

[77] Shin D, Blanchard SB, Ito M, Chu TM. Peripheral quantitative computer tomographic, histomorphometric, and removal torque analyses of two different non-coated implants in a rabbit model. Clin Oral Implants Res. 2011;22(3):242–50. 20831757 10.1111/j.1600-0501.2010.01980.x

[78] Schliephake H, Hefti T, Schlottig F, Gedet P, Staedt H. Mechanical anchorage and peri-implant bone formation of surface-modified zirconia in minipigs. J Clin Periodontol. 2010;37(9):818–28. 20573183 10.1111/j.1600-051X.2010.01549.x

[79] Sennerby L, Dasmah A, Larsson B, Iverhed M. Bone tissue responses to surface-modified zirconia implants: a histomorphometric and removal torque study in the rabbit. Clin Implant Dent Relat Res. 2005;7(Suppl 1):S13-20. 16137083 10.1111/j.1708-8208.2005.tb00070.x

[80] Nishihara H, Adanez MH, Att W. Current status of zirconia implants in dentistry: preclinical tests. J Prosthodont Res. 2019;63(1):1–14. 30205949 10.1016/j.jpor.2018.07.006

[81] Kaluderovic MR, Schreckenbach JP, Graf HL. Zirconia coated titanium for implants and their interactions with osteoblast cells. Mater Sci Eng C Mater Biol Appl. 2014;44:254–61. 25280704 10.1016/j.msec.2014.08.032

[82] da Cruz MB, Marques JF, Penarrieta-Juanito GM, Costa M, Souza JC, Magini RS, et al. Hard and soft tissue cell behavior on polyetheretherketone, zirconia, and titanium implant materials. Int J Oral Maxillofac Implants. 2019;34(1):39–46. 30282086 10.11607/jomi.6926

[83] Wei C, Gong T, Pow EHN, Botelho MG. Adhesive and oxidative response of stem cell and pre-osteoblasts on titanium and zirconia surfaces in vitro. J Investig Clin Dent. 2019;10(3): e12407. 30866178 10.1111/jicd.12407

[84] Gapski R, Martinez EF. Behavior of MC3T3-E1 osteoblastic cells cultured on titanium and zirconia surfaces: an in vitro study. Implant Dent. 2017;26(3):373–7. 28486352 10.1097/ID.0000000000000543

[85] Ogawa T, Nishimura I. Genes differentially expressed in titanium implant healing. J Dent Res. 2006;85(6):566–70. 16723657 10.1177/154405910608500617

[86] Kojima N, Ozawa S, Miyata Y, Hasegawa H, Tanaka Y, Ogawa T. High-throughput gene expression analysis in bone healing around titanium implants by DNA microarray. Clin Oral Implants Res. 2008;19(2):173–81. 18184341 10.1111/j.1600-0501.2007.01432.x

[87] Masuda T, Yliheikkila PK, Felton DA, Cooper LF. Generalizations regarding the process and phenomenon of osseointegration. Part I. In vivo studies. Int J Oral Maxillofac Implants. 1998;13(1):17–29. 9509776

[88] Dohan Ehrenfest DM, Vazquez L, Park YJ, Sammartino G, Bernard JP. Identification card and codification of the chemical and morphological characteristics of 14 dental implant surfaces. J Oral Implantol. 2011;37(5):525–42. 21728785 10.1563/AAID-JOI-D-11-00080

[89] Ikeda T, Hagiwara Y, Hirota M, Tabuchi M, Yamada M, Sugita Y, et al. Effect of photofunctionalization on fluoride-treated nanofeatured titanium. J Biomater Appl. 2014;28(8):1200–12. 23985537 10.1177/0885328213501566

[90] Nishimura I, Huang Y, Butz F, Ogawa T, Lin A, Wang CJ. Discrete deposition of hydroxyapatite nanoparticles on a titanium implant with predisposing substrate microtopography accelerated osseointegration. Nanotechnology. 2007;18(24):245101.

[91] Yamada M, Ueno T, Minamikawa H, Ikeda T, Nakagawa K, Ogawa T. Early-stage osseointegration capability of a submicrofeatured titanium surface created by microroughening and anodic oxidation. Clin Oral Implants Res. 2013;24(9):991–1001. 22726210 10.1111/j.1600-0501.2012.02507.x

[92] Ueno T, Tsukimura N, Yamada M, Ogawa T. Enhanced bone-integration capability of alkali- and heat-treated nanopolymorphic titanium in micro-to-nanoscale hierarchy. Biomaterials. 2011;32(30):7297–308. 21742375 10.1016/j.biomaterials.2011.06.033

[93] Tsukimura N, Ueno T, Iwasa F, Minamikawa H, Sugita Y, Ishizaki K, et al. Bone integration capability of alkali- and heat-treated nanobimorphic Ti-15Mo-5Zr-3Al. Acta Biomater. 2011;7(12):4267–77. 21888994 10.1016/j.actbio.2011.08.016

[94] Sugita Y, Ishizaki K, Iwasa F, Ueno T, Minamikawa H, Yamada M, et al. Effects of pico-to-nanometer-thin TiO2 coating on the biological properties of microroughened titanium. Biomaterials. 2011;32(33):8374–84. 21840046 10.1016/j.biomaterials.2011.07.077

[95] Ogawa T, Ozawa S, Shih JH, Ryu KH, Sukotjo C, Yang JM, et al. Biomechanical evaluation of osseous implants having different surface topographies in rats. J Dent Res. 2000;79(11):1857–63. 11145355 10.1177/00220345000790110701

[96] Ogawa T, Sukotjo C, Nishimura I. Modulated bone matrix-related gene expression is associated with differences in interfacial strength of different implant surface roughness. J Prosthodont. 2002;11(4):241–7. 12501137 10.1053/jpro.2002.129772

[97] Sato N, Kubo K, Yamada M, Hori N, Suzuki T, Maeda H, et al. Osteoblast mechanoresponses on Ti with different surface topographies. J Dent Res. 2009;88(9):812–6. 19767577 10.1177/0022034509343101

[98] Uno M, Ozawa R, Hamajima K, Saruta J, Ishigami H, Ogawa T. Variation in osteoblast retention ability of titanium surfaces with different topographies. J Dent Oral Biol. 2020;5(3):1169.

[99] Sykaras N, Iacopino AM, Marker VA, Triplett RG, Woody RD. Implant materials, designs, and surface topographies: their effect on osseointegration. A literature review. Int J Oral Maxillofac Implants. 2000;15(5):675–90. 11055135

[100] Wennerberg A, Albrektsson T. Suggested guidelines for the topographic evaluation of implant surfaces. Int J Oral Maxillofac Implants. 2000;15(3):331–44. 10874798

[101] Dalby MJ, Gadegaard N, Curtis AS, Oreffo RO. Nanotopographical control of human osteoprogenitor differentiation. Curr Stem Cell Res Ther. 2007;2(2):129–38. 18220898 10.2174/157488807780599220

[102] Uno M, Hayashi M, Ozawa R, Saruta J, Ishigami H, Ogawa T. Mechanical interlocking capacity of titanium with respect to surface morphology and topographical parameters. J Dent Oral Biol. 2020;5(2):1163.

[103] Aita H, Oh W, Kubo K, Tsukimura N, Maeda H, Ogawa T. Light-induced bone cement-philic titanium surface. J Mater Sci. 2008;43(5):1552–8.

[104] Klokkevold PR, Nishimura RD, Adachi M, Caputo A. Osseointegration enhanced by chemical etching of the titanium surface. A torque removal study in the rabbit. Clin Oral Implants Res. 1997;8(6):442–7. 9555202 10.1034/j.1600-0501.1997.080601.x

[105] Klokkevold PR, Johnson P, Dadgostari S, Caputo A, Davies JE, Nishimura RD. Early endosseous integration enhanced by dual acid etching of titanium: a torque removal study in the rabbit. Clin Oral Implants Res. 2001;12(4):350–7. 11488864 10.1034/j.1600-0501.2001.012004350.x

[106] Buser D, Nydegger T, Oxland T, Cochran DL, Schenk RK, Hirt HP, et al. Interface shear strength of titanium implants with a sandblasted and acid-etched surface: a biomechanical study in the maxilla of miniature pigs. J Biomed Mater Res. 1999;45(2):75–83. 10397960 10.1002/(sici)1097-4636(199905)45:2<75::aid-jbm1>3.0.co;2-p

[107] Wennerberg A, Albrektsson T, Andersson B. Bone tissue response to commercially pure titanium implants blasted with fine and coarse particles of aluminum oxide. Int J Oral Maxillofac Implants. 1996;11(1):38–45. 8820121

[108] Buser D, Nydegger T, Hirt HP, Cochran DL, Nolte LP. Removal torque values of titanium implants in the maxilla of miniature pigs. Int J Oral Maxillofac Implants. 1998;13(5):611–9. 9796144

[109] Buser D, Schenk RK, Steinemann S, Fiorellini JP, Fox CH, Stich H. Influence of surface characteristics on bone integration of titanium implants. A histomorphometric study in miniature pigs. J Biomed Mater Res. 1991;25(7):889–902. 1918105 10.1002/jbm.820250708

[110] Wennerberg A, Albrektsson T, Johansson C, Andersson B. Experimental study of turned and grit-blasted screw-shaped implants with special emphasis on effects of blasting material and surface topography. Biomaterials. 1996;17(1):15–22. 8962942 10.1016/0142-9612(96)80750-2

[111] Cochran DL, Schenk RK, Lussi A, Higginbottom FL, Buser D. Bone response to unloaded and loaded titanium implants with a sandblasted and acid-etched surface: a histometric study in the canine mandible. J Biomed Mater Res. 1998;40(1):1–11. 9511093 10.1002/(sici)1097-4636(199804)40:1<1::aid-jbm1>3.0.co;2-q

[112] Shalabi MM, Gortemaker A, Van’t Hof MA, Jansen JA, Creugers NH. Implant surface roughness and bone healing: a systematic review. J Dent Res. 2006;85(6):496–500. 16723643 10.1177/154405910608500603

[113] Stach RM, Kohles SS. A meta-analysis examining the clinical survivability of machined-surfaced and osseotite implants in poor-quality bone. Implant Dent. 2003;12(1):87–96. 12704962 10.1097/01.id.0000042507.37401.6f

[114] Nakamura H, Shim J, Butz F, Aita H, Gupta V, Ogawa T. Glycosaminoglycan degradation reduces mineralized tissue-titanium interfacial strength. J Biomed Mater Res A. 2006;77(3):478–86. 16482547 10.1002/jbm.a.30624

[115] Davies JE. Mechanisms of endosseous integration. Int J Prosthodont. 1998;11(5):391–401. 9922731

[116] Aita H, Att W, Ueno T, Yamada M, Hori N, Iwasa F, et al. Ultraviolet light-mediated photofunctionalization of titanium to promote human mesenchymal stem cell migration, attachment, proliferation and differentiation. Acta Biomater. 2009;5(8):3247–57. 19427421 10.1016/j.actbio.2009.04.022

[117] Takeuchi M, Abe Y, Yoshida Y, Nakayama Y, Okazaki M, Akagawa Y. Acid pretreatment of titanium implants. Biomaterials. 2003;24(10):1821–7. 12593964 10.1016/s0142-9612(02)00576-8

[118] Martin JY, Schwartz Z, Hummert TW, Schraub DM, Simpson J, Lankford J, et al. Effect of titanium surface roughness on proliferation, differentiation, and protein synthesis of human osteoblast-like cells (MG63). J Biomed Mater Res. 1995;29(3):389–401. 7542245 10.1002/jbm.820290314

[119] Komatsu K, Matsuura T, Suzumura T, Ogawa T. Genome-wide transcriptional responses of osteoblasts to different titanium surface topographies. Mater Today Bio. 2023;23: 100852. 38024842 10.1016/j.mtbio.2023.100852 PMC10663851

[120] Minamikawa H, Ikeda T, Att W, Hagiwara Y, Hirota M, Tabuchi M, et al. Photofunctionalization increases the bioactivity and osteoconductivity of the titanium alloy Ti6Al4V. J Biomed Mater Res A. 2014;102(10):3618–30. 24248891 10.1002/jbm.a.35030

[121] Mustafa K, Wennerberg A, Wroblewski J, Hultenby K, Lopez BS, Arvidson K. Determining optimal surface roughness of TiO(2) blasted titanium implant material for attachment, proliferation and differentiation of cells derived from human mandibular alveolar bone. Clin Oral Implants Res. 2001;12(5):515–25. 11564113 10.1034/j.1600-0501.2001.120513.x

[122] Schwartz Z, Raz P, Zhao G, Barak Y, Tauber M, Yao H, et al. Effect of micrometer-scale roughness of the surface of Ti6Al4V pedicle screws in vitro and in vivo. J Bone Joint Surg Am. 2008;90(11):2485–98. 18978418 10.2106/JBJS.G.00499 PMC2663328

[123] Malaval L, Liu F, Roche P, Aubin JE. Kinetics of osteoprogenitor proliferation and osteoblast differentiation in vitro. J Cell Biochem. 1999;74(4):616–27. 10440931

[124] Marchisio M, Di Carmine M, Pagone R, Piattelli A, Miscia S. Implant surface roughness influences osteoclast proliferation and differentiation. J Biomed Mater Res B Appl Biomater. 2005;75(2):251–6. 16078239 10.1002/jbm.b.30287

[125] Thomas DM, Johnson SA, Sims NA, Trivett MK, Slavin JL, Rubin BP, et al. Terminal osteoblast differentiation, mediated by runx2 and p27KIP1, is disrupted in osteosarcoma. J Cell Biol. 2004;167(5):925–34. 15583032 10.1083/jcb.200409187 PMC2172443

[126] Hori N, Iwasa F, Tsukimura N, Sugita Y, Ueno T, Kojima N, et al. Effects of UV photofunctionalization on the nanotopography enhanced initial bioactivity of titanium. Acta Biomater. 2011;7(10):3679–91. 21723964 10.1016/j.actbio.2011.06.022

[127] Tsukimura N, Yamada M, Iwasa F, Minamikawa H, Att W, Ueno T, et al. Synergistic effects of UV photofunctionalization and micro-nano hybrid topography on the biological properties of titanium. Biomaterials. 2011;32(19):4358–68. 21421270 10.1016/j.biomaterials.2011.03.001

[128] Yamada M, Ueno T, Tsukimura N, Ikeda T, Nakagawa K, Hori N, et al. Bone integration capability of nanopolymorphic crystalline hydroxyapatite coated on titanium implants. Int J Nanomed. 2012;7:859–73. 10.2147/IJN.S28082 PMC3284227 22359461

[129] Meirelles L, Arvidsson A, Andersson M, Kjellin P, Albrektsson T, Wennerberg A. Nano hydroxyapatite structures influence early bone formation. J Biomed Mater Res A. 2008;87(2):299–307. 18181110 10.1002/jbm.a.31744

[130] Iwasa F, Tsukimura N, Sugita Y, Kanuru RK, Kubo K, Hasnain H, et al. TiO2 micro-nano-hybrid surface to alleviate biological aging of UV-photofunctionalized titanium. Int J Nanomed. 2011;6:1327–41. 10.2147/IJN.S22099 PMC3133524 21760728

[131] Hirota M, Hori N, Sugita Y, Ikeda T, Park W, Saruta J, et al. A novel cell delivery system exploiting synergy between fresh titanium and fibronectin. Cells. 2022;11(14):2158. 35883601 10.3390/cells11142158 PMC9317518

[132] Kido D, Komatsu K, Suzumura T, Matsuura T, Cheng J, Kim J, et al. Influence of surface contaminants and hydrocarbon pellicle on the results of wettability measurements of titanium. Int J Mol Sci. 2023;24(19):14688. 37834133 10.3390/ijms241914688 PMC10572547

[133] Komatsu K, Chao D, Matsuura T, Kido D, Ogawa T. Advancing osseointegration research: a dynamic three-dimensional (3D) in vitro culture model for dental implants. J Dent Sci. 2025;20(1):350–60. 39873044 10.1016/j.jds.2024.06.018 PMC11763192

[134] Hori N, Att W, Ueno T, Sato N, Yamada M, Saruwatari L, et al. Age-dependent degradation of the protein adsorption capacity of titanium. J Dent Res. 2009;88(7):663–7. 19641155 10.1177/0022034509339567

[135] Ogawa T. UV-photofunctionalization of titanium implants. Oral Craniofacial Tissue Eng. 2012;2:151–8.

[136] Weinlaender M, Kenney EB, Lekovic V, Beumer J 3rd, Moy PK, Lewis S. Histomorphometry of bone apposition around three types of endosseous dental implants. Int J Oral Maxillofac Implants. 1992;7(4):491–6. 1299645

[137] Berglundh T, Abrahamsson I, Albouy JP, Lindhe J. Bone healing at implants with a fluoride-modified surface: an experimental study in dogs. Clin Oral Implants Res. 2007;18(2):147–52. 17269959 10.1111/j.1600-0501.2006.01309.x

[138] De Maeztu MA, Braceras I, Alava JI, Gay-Escoda C. Improvement of osseointegration of titanium dental implant surfaces modified with CO ions: a comparative histomorphometric study in beagle dogs. Int J Oral Maxillofac Surg. 2008;37(5):441–7. 18339518 10.1016/j.ijom.2008.01.010

[139] Suzuki T, Hori N, Att W, Kubo K, Iwasa F, Ueno T, et al. Ultraviolet treatment overcomes time-related degrading bioactivity of titanium. Tissue Eng Part A. 2009;15(12):3679–88. 19397472 10.1089/ten.TEA.2008.0568

[140] Suzuki T, Kubo K, Hori N, Yamada M, Kojima N, Sugita Y, et al. Nonvolatile buffer coating of titanium to prevent its biological aging and for drug delivery. Biomaterials. 2010;31(18):4818–28. 20350765 10.1016/j.biomaterials.2010.02.061

[141] Suzuki S, Kobayashi H, Ogawa T. Implant stability change and osseointegration speed of immediately loaded photofunctionalized implants. Implant Dent. 2013;22(5):481–90. 24021973 10.1097/ID.0b013e31829deb62

[142] Hirota M, Ozawa T, Iwai T, Mitsudo K, Ogawa T. UV-mediated photofunctionalization of dental implant: a seven-year results of a prospective study. J Clin Med. 2020;9(9):2733. 32847061 10.3390/jcm9092733 PMC7565265

[143] Hirota M, Ozawa T, Iwai T, Ogawa T, Tohnai I. Implant stability development of photofunctionalized implants placed in regular and complex cases: a case-control study. Int J Oral Maxillofac Implants. 2016;31(3):676–86. 27183088 10.11607/jomi.4115

[144] Hirota M, Ozawa T, Iwai T, Ogawa T, Tohnai I. Effect of photofunctionalization on early implant failure. Int J Oral Maxillofac Implants. 2018;33(5):1098–102. 30231097 10.11607/jomi.6541

[145] Komatsu K, Matsuura T, Suzumura T, Shibata R, Chen PC, Ogawa T. Vacuum Ultraviolet (VUV)-induced physicochemical engineering of titanium: enhanced fibroblast activity, redox system, and glycosaminoglycan binding for soft tissue integration. ACS Appl Bio Mater. 2025;8(5):4166–85. 40249645 10.1021/acsabm.5c00283

[146] Matsuura T, Komatsu K, Suzumura T, Stavrou S, Juanatas ML, Park W, et al. Enhanced functionality and migration of human gingival fibroblasts on vacuum ultraviolet light-treated titanium: an implication for mitigating cellular stress to improve peri-implant cellular reaction. J Prosthodont Res. 2025;69(2):249–58. 39198200 10.2186/jpr.JPR_D_24_00071

[147] Suzumura T, Matsuura T, Komatsu K, Ogawa T. Decomposing organic molecules on titanium with vacuum ultraviolet light for effective and rapid photofunctionalization. J Funct Biomater. 2022;14(1):11. 36662058 10.3390/jfb14010011 PMC9861116

[148] Klinger A, Steinberg D, Kohavi D, Sela MN. Mechanism of adsorption of human albumin to titanium in vitro. J Biomed Mater Res. 1997;36(3):387–92. 9260109 10.1002/(sici)1097-4636(19970905)36:3<387::aid-jbm13>3.0.co;2-b

[149] Ellingsen JE. A study on the mechanism of protein adsorption to TiO2. Biomaterials. 1991;12(6):593–6. 1663394 10.1016/0142-9612(91)90057-h

[150] Steinberg D, Klinger A, Kohavi D, Sela MN. Adsorption of human salivary proteins to titanium powder. I. Adsorption of human salivary albumin. Biomaterials. 1995;16(17):1339–43. 8573673 10.1016/0142-9612(95)91050-9

[151] Iwasa F, Hori N, Ueno T, Minamikawa H, Yamada M, Ogawa T. Enhancement of osteoblast adhesion to UV-photofunctionalized titanium via an electrostatic mechanism. Biomaterials. 2010;31(10):2717–27. 20035996 10.1016/j.biomaterials.2009.12.024

[152] Kyte J. The basis of the hydrophobic effect. Biophys Chem. 2003;100(1–3):193–203. 12646366 10.1016/s0301-4622(02)00281-8

[153] Minamikawa H, Att W, Ikeda T, Hirota M, Ogawa T. Long-term progressive degradation of the biological capability of titanium. Materials. 2016;9:102. 10.3390/ma9020102. 28787899 10.3390/ma9020102 PMC5456510

[154] Kitajima H, Hirota M, Osawa K, Iwai T, Saruta J, Mitsudo K, et al. Optimization of blood and protein flow around superhydrophilic implant surfaces by promoting contact hemodynamics. J Prosthodont Res. 2022;67(4):568–82. 36543189 10.2186/jpr.JPR_D_22_00225

[155] Kitajima H, Hirota M, Iwai T, Hamajima K, Ozawa R, Hayashi Y, et al. Computational fluid simulation of fibrinogen around dental implant surfaces. Int J Mol Sci. 2020;21(2):660. 31963895 10.3390/ijms21020660 PMC7014059

[156] Kitajima H, Hirota M, Osawa K, Iwai T, Mitsudo K, Saruta J, et al. The effects of a biomimetic hybrid meso- and nano-scale surface topography on blood and protein recruitment in a computational fluid dynamics implant model. Biomimetics (Basel). 2023;8(4):376. 37622981 10.3390/biomimetics8040376 PMC10452410

[157] Kitajima H, Hirota M, Iwai T, Mitsudo K, Saruta J, Ogawa T. Synergistic enhancement of protein recruitment and retention via implant surface microtopography and superhydrophilicity in a computational fluid dynamics model. Int J Mol Sci. 2023;24:15618. 37958605 10.3390/ijms242115618 PMC10649348

[158] Hayashi R, Ueno T, Migita S, Tsutsumi Y, Doi H, Ogawa T, et al. Hydrocarbon deposition attenuates osteoblast activity on titanium. J Dent Res. 2014;93(7):698–703. 24868012 10.1177/0022034514536578 PMC4293731

[159] Ghassemi A, Ishijima M, Hasegawa M, Mohammadzadeh Rezaei N, Nakhaei K, Sekiya T, et al. Biological and physicochemical characteristics of 2 different hydrophilic surfaces created by saline-storage and ultraviolet treatment. Implant Dent. 2018;27(4):405–14. 29851661 10.1097/ID.0000000000000773

[160] Ueno T, Takeuchi M, Hori N, Iwasa F, Minamikawa H, Igarashi Y, et al. Gamma ray treatment enhances bioactivity and osseointegration capability of titanium. J Biomed Mater Res B Appl Biomater. 2012;100(8):2279–87. 22987777 10.1002/jbm.b.32799

[161] Suzumura T, Matsuura T, Komatsu K, Ogawa T. A novel high-energy vacuum ultraviolet light photofunctionalization approach for decomposing organic molecules around titanium. Int J Mol Sci. 2023;24(3):1978. 36768297 10.3390/ijms24031978 PMC9916712

[162] Kitajima H, Hirota M, Komatsu K, Isono H, Matsuura T, Mitsudo K, et al. Ultraviolet light treatment of titanium microfiber scaffolds enhances osteoblast recruitment and osteoconductivity in a vertical bone augmentation model: 3D UV photofunctionalization. Cells. 2023;12(1):19. 10.3390/cells12010019 PMC9818481 36611812

[163] Hirota M, Ikeda T, Sugita Y, Ishijima M, Hirota S, Ogawa T. Impaired osteoblastic behavior and function on saliva-contaminated titanium and its restoration by UV treatment. Mater Sci Eng C Mater Biol Appl. 2019;100:165–77. 30948050 10.1016/j.msec.2019.03.008

[164] Ding X, Xu S, Li S, Guo Z, Lu H, Lai C, et al. Biological effects of titanium surface charge with a focus on protein adsorption. ACS Omega. 2020;5(40):25617–24. 33073087 10.1021/acsomega.0c02518 PMC7557225

[165] Hori N, Ueno T, Minamikawa H, Iwasa F, Yoshino F, Kimoto K, et al. Electrostatic control of protein adsorption on UV-photofunctionalized titanium. Acta Biomater. 2010;6(10):4175–80. 20466081 10.1016/j.actbio.2010.05.006

[166] Ikeda T, Okubo T, Saruta J, Hirota M, Kitajima H, Yanagisawa N, et al. Osteoblast attachment compromised by high and low temperature of titanium and its restoration by UV photofunctionalization. Materials (Basel). 2021;14(19):5493. 34639891 10.3390/ma14195493 PMC8509491

[167] Soltanzadeh P, Ghassemi A, Ishijima M, Tanaka M, Park W, Iwasaki C, et al. Success rate and strength of osseointegration of immediately loaded UV-photofunctionalized implants in a rat model. J Prosthet Dent. 2017;118(3):357–62. 28222880 10.1016/j.prosdent.2016.11.008

[168] Minamikawa H, Att W, Ikeda T, Hirota M, Ogawa T. Long-term progressive degradation of the biological capability of titanium. Materials (Basel). 2016;9(2):102. 28787899 10.3390/ma9020102 PMC5456510

[169] Ueno T, Yamada M, Suzuki T, Minamikawa H, Sato N, Hori N, et al. Enhancement of bone-titanium integration profile with UV-photofunctionalized titanium in a gap healing model. Biomaterials. 2010;31(7):1546–57. 19962757 10.1016/j.biomaterials.2009.11.018

[170] Yamazaki M, Yamada M, Ishizaki K, Sakurai K. Ultraviolet-C irradiation to titanium implants increases peri-implant bone formation without impeding mineralization in a rabbit femur model. Acta Odontol Scand. 2015;73(4):302–11. 25645878 10.3109/00016357.2014.956332

[171] Lee JB, Jo YH, Choi JY, Seol YJ, Lee YM, Ku Y, et al. The effect of ultraviolet photofunctionalization on a titanium dental implant with machined surface: an in vitro and in vivo study. Materials (Basel). 2019;12(13):2078. 31261627 10.3390/ma12132078 PMC6650865

[172] Ueno T, Yamada M, Hori N, Suzuki T, Ogawa T. Effect of ultraviolet photoactivation of titanium on osseointegration in a rat model. Int J Oral Maxillofac Implants. 2010;25(2):287–94. 20369086

[173] Dini C, Nagay BE, Magno MB, Maia LC, Barao VAR. Photofunctionalization as a suitable approach to improve the osseointegration of implants in animal models-A systematic review and meta-analysis. Clin Oral Implants Res. 2020;31(9):785–802. 32564392 10.1111/clr.13627

[174] Lang X, Qiao B, Ge Z, Yan J, Zhang Y. Clinical effects of photofunctionalization on implant stability and marginal bone loss: systematic review and meta-analysis. J Clin Med. 2022;11(23):7042. 36498616 10.3390/jcm11237042 PMC9739233

[175] Chang LC. Clinical applications of photofunctionalization on dental implant surfaces: a narrative review. J Clin Med. 2022;11(19):5823. 36233693 10.3390/jcm11195823 PMC9571244

[176] Suzumura T, Matusura T, Komatsu K, Sugita Y, Maeda H, Ogawa T. Vacuum ultraviolet (VUV) light photofunctionalization to induce human oral fibroblast transmigration on zirconia. Cells. 2023;12:2542. 37947620 10.3390/cells12212542 PMC10647316

